# Review on Recent Progress in Magnetic Nanoparticles: Synthesis, Characterization, and Diverse Applications

**DOI:** 10.3389/fchem.2021.629054

**Published:** 2021-07-13

**Authors:** Arbab Ali, Tufail Shah, Rehmat Ullah, Pingfan Zhou, Manlin Guo, Muhammad Ovais, Zhiqiang Tan, YuKui Rui

**Affiliations:** ^1^ Beijing Key Laboratory of Farmland Soil Pollution Prevention and Remediation, College of Resources and Environmental Sciences, China Agricultural University, Beijing, China; ^2^ CAS Key Laboratory for Biomedical Effects of Nanomaterials and Nanosafety, CAS Center for Excellence in Nanoscience, National Center for Nanoscience and Technology (NCNST), Beijing, China; ^3^ College of Land Science and Technology, China Agricultural University, Beijing, China; ^4^ Key Laboratory of Crop Heterosis and Utilization (MOE)/Beijing Key Laboratory of Crop Genetic Improvement, College of Agronomy and Biotechnology, China Agricultural University, Beijing, China; ^5^ State Key Laboratory of Environmental Chemistry and Ecotoxicology, Research Center for Eco-Environmental Sciences, Chinese Academy of Sciences, Beijing, China

**Keywords:** magnetic nanoparticles, synthesis, characterization, composition, applications

## Abstract

Diverse applications of nanoparticles (NPs) have revolutionized various sectors in society. In the recent decade, particularly magnetic nanoparticles (MNPs) have gained enormous interest owing to their applications in specialized areas such as medicine, cancer theranostics, biosensing, catalysis, agriculture, and the environment. Controlled surface engineering for the design of multi-functional MNPs is vital for achieving desired application. The MNPs have demonstrated great efficacy as thermoelectric materials, imaging agents, drug delivery vehicles, and biosensors. In the present review, first we have briefly discussed main synthetic methods of MNPs, followed by their characterizations and composition. Then we have discussed the potential applications of MNPs in different with representative examples. At the end, we gave an overview on the current challenges and future prospects of MNPs. This comprehensive review not only provides the mechanistic insight into the synthesis, functionalization, and application of MNPs but also outlines the limits and potential prospects.

**GRAPHICAL ABSTRACT:**
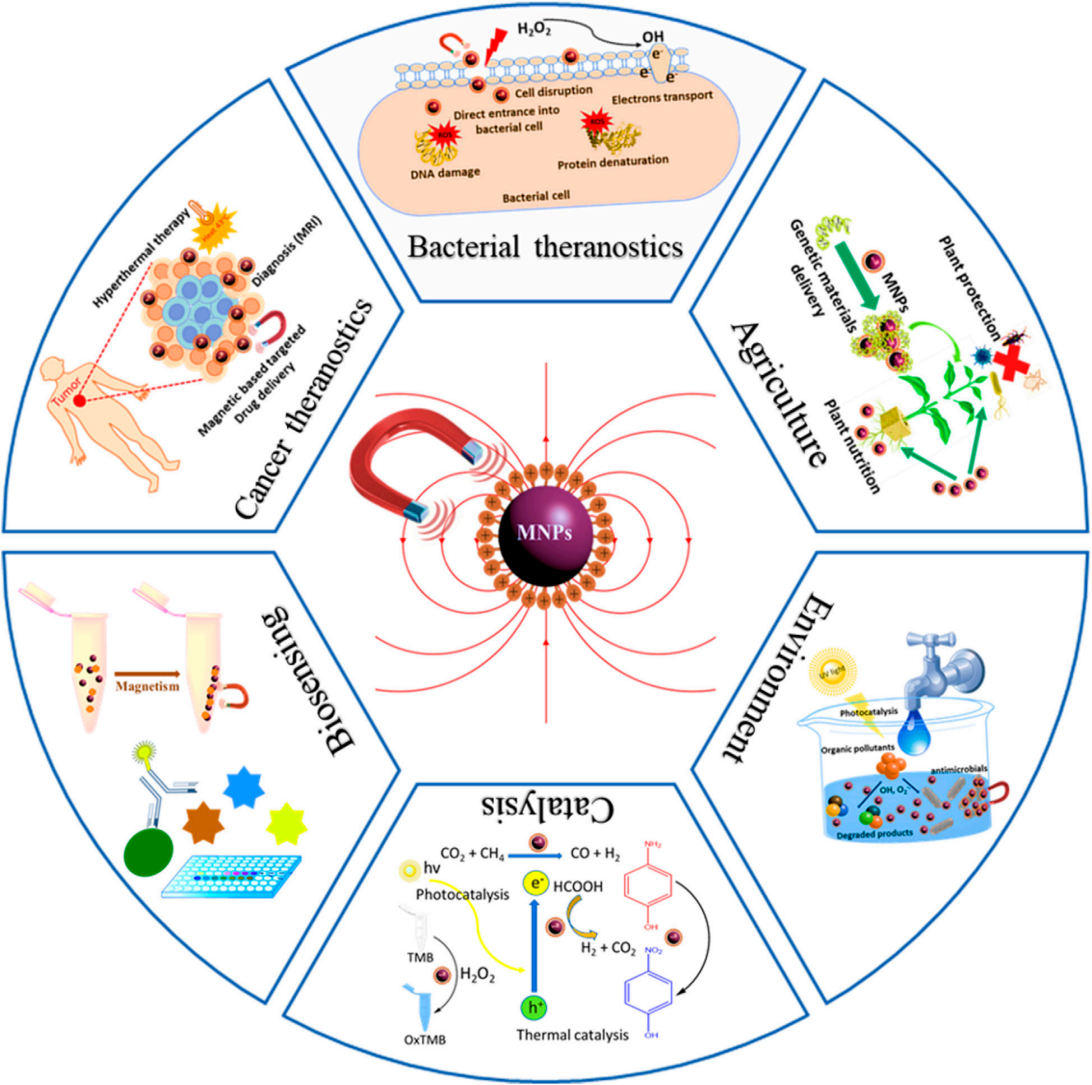


## Introduction

Recently, the advancement witnessed in the nanotechnology field has contributed to the improvement and revolutionizing of different fields. The list of benefits and applications of nanotechnology is growing rapidly. Nanoparticles (NPs) are small particles that exist in an average size ranges between 1 and 100 nm that distinguish them from their parental bulky materials and make them ideal for diverse applications (Laconte et al., 2005; Cardoso et al., 2018). Among them, magnetic nanoparticles (MNPs), a nanoscale material, with unique magnetic properties have been widely used in different fields such as biomedical, energy, engineering, and environment applications. The MNPs have become an area of intensive research in the recent past because of their unique and distinguished properties which make their potential application in biomedicine, catalysis, agriculture, and environment (Hao et al., 2010; Wu et al., 2016; Thorat et al., 2017; Zhu et al., 2018; Zhang et al., 2019). The MNPs are formed from different metal elements (either alone or in composites) and their oxides with magnetic characteristics (Kefeni et al., 2017). Especially, the superparamagnetic magnetite (Fe_3_O_4_) is the most commonly used iron oxide because of its high biocompatibility and low toxicity (Assa et al., 2016; Farjadian et al., 2017). Recently, iron oxide MNPs are receiving tremendous attention in developing and understanding their applicability in multiple areas (Bansal et al., 2017). Iron oxide MNPs with smaller sizes are the best choices for biological and biomedical applications (Lu et al., 2017; Mohammed et al., 2017). The surface chemistry of superparamagnetic iron oxides MNPs can be controlled by altering its physicochemical properties and applied in different fields e.g., Hyperthermia, magnetic resonance imaging (MRI), immunoassays, drug, and cell separation (Weissleder et al., 1995; Kumar and Mohammad, 2011). Properties like high surface area, metal-rich moieties, and tunable structures make MNPs a field of great interest with broad application in environmental, biomedical, catalysis, drug delivery, and bioimaging. Their tuning properties of shape and size have made them a hot topic in the past decades (Singamaneni et al., 2011; Purbia and Paria, 2015). The recent development and an unprecedented number of publications have made MNPs a subject of utmost importance and became an emerging field of nano science and nanobiotechnology (Kudr et al., 2017; Cardoso et al., 2018; Wu et al., 2019). The physicochemical properties of MNPs are different from their parental bulky material in terms of large specific surface area, which makes them more superparamagnetic (Babes et al., 1999). Nanomaterials with well-defined structures are critical to achieve these properties (Issa et al., 2013). In synthesizing monodisperse magnetic nanostructures, there are some challenges like dipolar interactions, particle surface effects, and size control, etc. are of great concern. However, some innovative chemical synthetic approaches have made it easier to restrict the nucleation and growth of these MNPs. Thus, the size and shape of MNPs are much controllable and highly depends on the type of surfactant, solvents used under different reaction conditions (Xie et al., 2018). Keeping in mind the current interest in MNPs, this review is constructed by summarizing the present and past studies with recent developments in the synthesis of MNPs with their respective advantages and disadvantages. This review is designed to report information regarding different classification of MNPs based on different elemental compositions and metals followed by their characterization and applications in the fields of energy, biomedicine, biosensing, environmental, agriculture, and catalysis, cancer. In addition, the future challenges associated with MNPs are also discussed in later section.

## Main Synthesis Methods of MNPs

**FIGURE 1:**
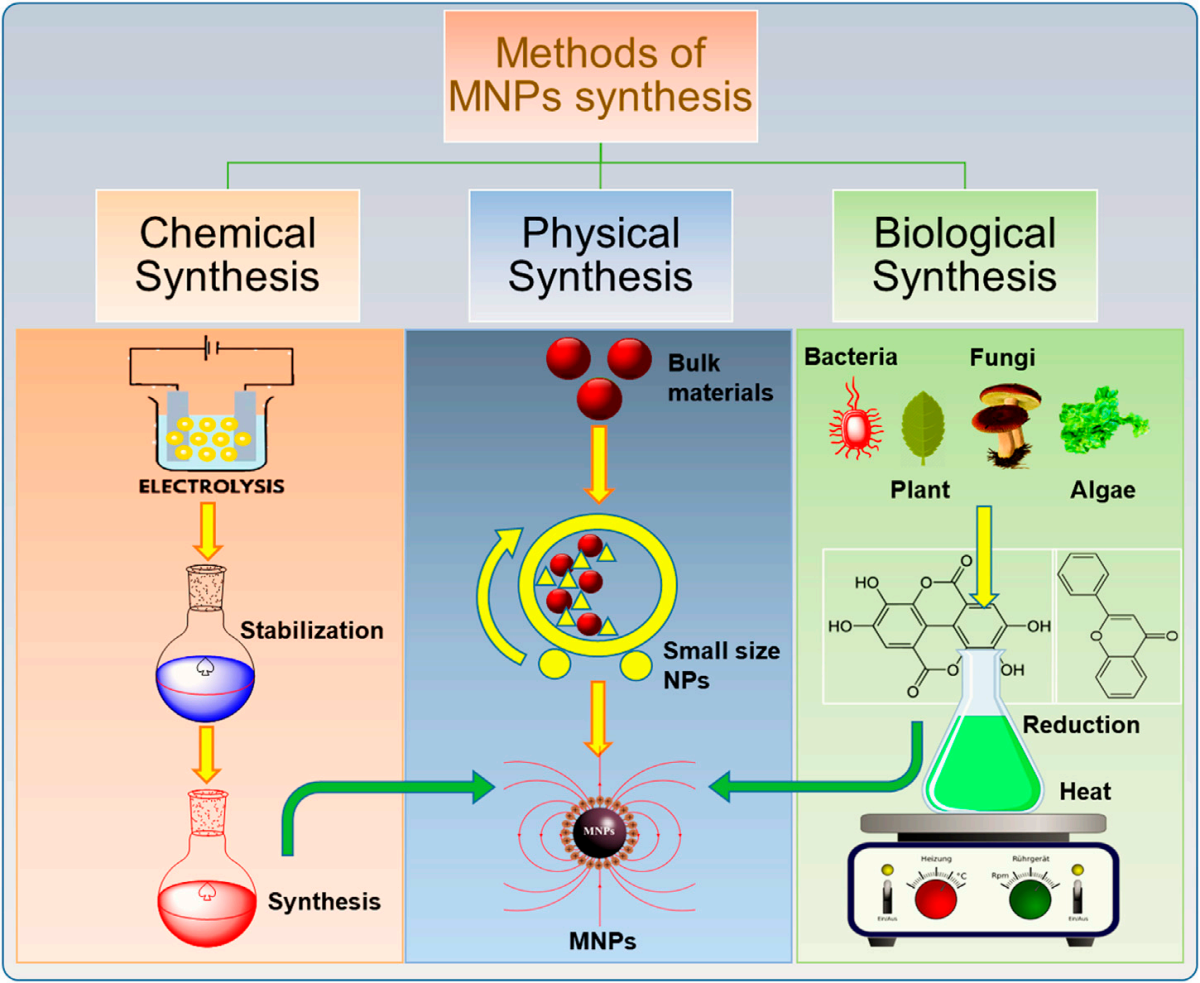
Schematic illustration of the synthesis of magnetic nanoparticles (MNPs) with different methods.

The past decade has witnessed extensive research in the development of different approaches for the synthesis MNPs. Different synthetic methods are used to obtain MNPs of desired size, morphology, stability, and biocompatibility. The most common methods include the ball milling method, coprecipitation, thermal decomposition, hydrothermal, microemulsion, sol-gel method, and biological method to produce MNPs. A graphical illustration of MNPs prepared through various routes (physical, chemical, and biological) are given in Figure 1.

### Physical Methods

The physical methods consist of “top-down” and “bottom-up” approaches. In the top-down approach, the bulk materials are broken into nano-sized particles i.e., through high energy ball milling. It is difficult to obtain NPs of desired shape and size through mechanical crushing (Decastro and Mitchell, 2002). While in bottom-up approach, the well-dispersed and fine nano-scaled tiny particles can be obtained than the top-down approach. The example of bottom-up approach is laser evaporation (Biehl et al., 2018). Some other physical methods like wire explosion method and inert-gas condensation method are also used to prepare MNPs. In this review, we will discuss three physical methods i.e., ball milling, laser evaporation, and wire explosion method.

#### Ball Milling Method/Mechanical Method

Ball milling is a top-down approach of producing MNPs from the bulk material. It is a simple and convenient process which involves the mechanical grinding of coarse-textured particles into fine-textured particles (Fecht et al., 1990; El-Eskandarany, 2001). This method was first developed in 1970 (Benjamin, 1970). The working principle is very simple; the raw materials are enclosed in a small hollow cylindrical jar containing many steel balls as a grinding medium. The balls apply kinetic energy to the solid material as a result of continuous collisions between steel balls and solid materials which results in nano/micro-sized powder. The ball to powder ratio, ball size, vibration speed, and milling time are the main factors that affect the formation process of nano/micro size crystals. The main disadvantage of this process is the contamination of the product (Mohamed and Mohamed, 2019). The particles have wide size distribution as compared to synthesized by chemical methods.

#### Laser Evaporation

Laser evaporation is a bottom-up approach in which nanoparticles are formed through condensation from liquid or gaseous phase (Biehl et al., 2018). The laser evaporation also called laser ablation is a simple technique in which high energy laser is applied for production of MNPs. This method is also suitable for producing iron oxide MNPs (Shin et al., 2004). In this process, particles of coarse textured (in µm or mm size ranges) are selected as raw materials and are evaporated through under the focus of laser beam. The material is placed at the bottom of a cell submerged in a liquid solution and targeted by the focused laser beam. The irradiation of the material in a solution takes place by a laser beam. The vapors of the material are cooled down in a gas phase and as a result a fast condensation and nucleation takes place that lead to the formation of nanoparticles (Kurland et al., 2007). This method is low cost-effective and it do not require any expensive chemical or produce any hazardous waste as in wet chemistry methods (Yang, 2007; Amendola and Meneghetti, 2009).

#### Wire Explosion Method

The wire explosion technique is a new physiochemical technique which is a safe and clean process for synthesizing MNPs. This method is a one-step highly productive process which requires no additional steps like separation of NPs from solution and re-treatment of byproducts. This method was previously used to prepare iron oxide MNPs for removal of arsenic from water (Song et al., 2013). It is environmentally safe and requires minimum energy for making less contaminated nano powders (Kotov, 2003). The NPs produced through this method are not monodispersed (Kawamura et al., 2015).

### Chemical Methods

The chemical synthesis methods consist of different bottom-up approaches. A comprehensive description of some common methods that are widely used to synthesize MNPs is provided below.

#### Coprecipitation Method

Coprecipitation is the most commonly used method for producing MNPs of controlled size and magnetic properties (Sandeep Kumar, 2013). It includes the use of less harmful materials and procedures, and widely practiced in biomedical applications (Indira and Lakshmi, 2010). The synthesis of MNPs through coprecipitation is very convenient and facile when we need nanocrystals in large quantities. This method is very common for the production of NPs of controlled size with good magnetic properties. Different metal ions are used to dissolve in a solvent to produce MNPs. NPs of manganese ferrite (MnFe_2_O_4_) were formed by using ferric chloride (FeCl_3_)and manganese(II) chloride (MnCl_2_) as the metal ions and salts of sodium hydroxide (NaOH) as precipitant (Chen et al., 1996). The nanocrystals of MgFe_2_SO_4_ can be formed by Fe^3+^ and Mg^2+^ ions, coprecipitated by adding NaOH (Chen et al., 1999). Similarly, in another study, Fe^2+^ and Fe^3+^ ions are coprecipitated to get Fe_3_O_4_ NPs (Shen et al., 2014; Mireles et al., 2016). During the process of coprecipitation, different factors like pH, metal ions, and their concentrations, the nature of salt, the reaction temperature can affect the composition of MNPs, particle size, and shape (Mosayebi et al., 2017). The MNPs synthesis through coprecipitation is quite simple to obtain uniformly dispersed small size NPs (Jiang et al., 2004). Moreover, this method is preferred because of its simplicity but, sometimes it is difficult to control the shape of MNPs via coprecipitation.

#### Thermal Decomposition Method

In this method, organometallic precursors are used to produce monodispersed NPs under extreme temperature. The MNPs prepared by this method have high crystallinity, controlled size and well-defined shape. The process of decomposition of organometallic precursors is carried out under the presence of organic surfactants to produce MNPs of desired size and shape (Effenberger et al., 2017). The stabilizing agents used for the synthesis of MNPs include fatty acids, hexadecylamine, and oleic acid. The stabilizers used in the decomposition process can slow down the nucleation of NPs which control the growth of MNPS and help in producing a spherical shape and desirable size of less than 30 nm. The nanocrystals of Fe_3_O_4_ and magnetically active composites of iron were reported to produce by this approach (Jana et al., 2004; Ren et al., 2019). The thermal decomposition of zero-valent metal precursor Fe (CO)_5_ leads to the formation of metal NPs, but if oxidation occurs it may form iron oxide MNPs of high quality. While on the other hand if the decomposition of precursors occurs with cationic metal centers can result in the direct formation of metal oxide NPs (Frey et al., 2009; Effenberger et al., 2017). Monodispersed iron oxide MNPs ranging from 6 to 20 nm were previously synthesized through polymer-catalyzed decomposition of Fe(CO)_5_ (Smith and Wychick, 1980; Dale and Huber, 2005). The temperature requirement depends on the type of precursor used. The degree of temperature, reaction time, type of surfactants and solvents, and aging period is adjusted according to the desired shape and size (Lu et al., 2007; Patsula et al., 2016). To date, this method has been reported as one of the best methods to produce MNPs on a large scale in uniform size and homogeneous shape (Kudr et al., 2017). The risk factor associated with this method is the production of toxic organic-soluble solvents, which limits its application in the biomedical field (Faraji et al., 2010). The thermal composition is comparatively more useful than coprecipitation for synthesizing magnetic particles of smaller size.

#### Microemulsion Synthesis Method

Microemulsions are turbid systems of lipophilic and hydrophilic phases that involve surfactants or sometimes co-surfactants. This is an isotropic transparent liquid system of water, oil, and amphiphile. In this process, oil is mixed with a surfactant and water is magnetically stirred at ambient temperature. There are three kinds of microemulsions; 1) oil in water (O/W), which is the aqueous phase with some oil droplets, 2) water in oil (W/O), which is oil as a dominant phase with some droplets of water, 3) Both oil and water are present in a comparable amount. For example, microemulsion of w/o type, droplets of water in organic solvent were coated by a surfactant reducing the size MNPs (Lopez Perez et al., 1997; Mosayebi et al., 2017). The shape and size of MNPs prepared through this method depends on what kind of surfactant is used (Lu et al., 2013). Some iron oxide MNPs were prepared through the w/o type of microemulsion, in which they used two microdroplets, one with metal percussor and another with a precipitating agent (Okoli et al., 2011). This method was followed to prepare MNPS with silica-coating and were further modified with amino, which was useful for tumor cell separation (Zhang et al., 2016). The MNPs prepared by microemulsion are of low quantity and uniformly dispersed.

#### Hydrothermal Synthesis Method

This method is used to prepare NPs in an aqueous solution, under high pressure and temperature (Zhang et al., 2016). Hydrothermal also referred to as solvothermal is one of the successful solution reaction-based approaches through which MNPs are produced at high pressure and temperature. Under the hydrothermal process, hydrolysis and oxidation reaction takes place to produce MNPs (Reddy et al., 2012). The crystal formation depends on the extent of the solubility of minerals in the water. Particles of various magnetic nanomaterials of uniform size were obtained through this method (Wang et al., 2005). For example, Fe_3_O_4_ NPs of size 15 nm and spherical shape were obtained and applied in the tumor MRI (Li et al., 2013). Similarly, Chitosan-coated Fe_3_O_4_ NPs of 25 nm size were prepared and applied in enzyme immobilization (Li et al., 2008). The morphology and crystallinity of synthesized MNPs depend on the appropriate mixing of solvent, time, amount of pressure, and temperature. Following this approach can yield more NPs as compared to the microemulsion method. But this process needs high temperature and pressure, therefore, it is done with great care and carried out in a special equipment. Comparatively, hydrothermal method is preferred over sol-gel and other methods because of its advantages of producing NPs of desirable shape, size, with high crystallinity and consistent composition (Zahid et al., 2019).

#### Sol-Gel Method

The whole chemistry in this process involves gel formation at room temperature by hydrolysis and polycondensation reactions of metal alkoxides. Metallic salts are dissolved in water or other solvents and are homogeneously dispersed to prepare sol or colloidal solution (Ansari et al., 2019). The van der Waals forces between the particles occur and the interaction between particles increases by stirring and increasing the temperature. The mixture is heated until the solvent is removed and the solution is dried, which finally results in the formation of gel (Hasany et al., 2012; Mohamed and Mohamed, 2019). This method is useful in the production of iron oxide MNPs and silica-coated MNPs. The MNPs can be produced in large quantities with control size and well-defined shape (Lu et al., 2007). Previously, MNPs were prepared by heating the mixture of FeCl_3_ and NaOH solution at 50–100°C (Gu et al., 2006). For the sol-gel method, no special equipment is needed and can be done at room temperature which makes it a cheaper technology. This method is very simple in controlling the composition, shape, and size of MNPs. The solid material produced through this method are highly pure with good crystallinity and tunability. However, in some cases, this method results in contamination from byproduct reactions, and the thus need to be retreated to obtain pure MNPs. The generation of three-dimensional oxide networks limits its efficiency in the production of dispersed NPs (Hasany et al., 2012). Other disadvantages of this method is to require prolong reaction time and involve toxic organic solvents.

### Biological Synthesis Method

Biological synthesis is well-known method to synthesize MNPs by using living organisms like plants and microorganisms (fungi, viruses, bacteria, and actinomycetes) (Verma et al., 2021). The MNPs produced this method are comparatively biocompatible and useful application in the biomedical field. The benefits of this method are its efficiency, ecofriendly, and clean process. While the disadvantage is its poor dispersion of the NPs (Komeili, 2012). The synthesis of NPs by using plant tissue, extracts, exudates, and other parts of the plant has become an area of great interest for researchers (Gul et al., 2019). For example, particles with an average size of 60 nm ferromagnetic magnetite were reported to biologically synthesize (Lenders et al., 2014). Biological synthesis is a promising technique that emerged in recent years, but the mechanism of formation of NPs by using microorganisms and plants is not well understood and still under investigation (Komeili, 2012; Duan et al., 2018). For example, some investigations suggested possible mechanisms for the mycosynthesis of metal NPs. Three mechanisms were suggested; 1) activity of nitrate reductase, 2) shuttle electrons quinones, and 3) mixed mechanism. However, the mechanism is not very clear to acknowledge so far as to prepare MNPs (Komeili, 2012). Biologically synthesized Fe_3_O_4_ magnetic material was used in Suzuki-Miyaura reaction and photo-catalysis as a catalyst (Zhang et al., 2019). Some shortcomings associated with this method like yield and MNPs dispersion still need to be investigated (Lu et al., 2007; Duan et al., 2018).

### Comparison of Different Synthesis Methods

Different techniques have been developed for the synthesis of MNPs. These synthetic approaches are categorized into three different methods, i.e., physical methods, chemical methods, and biological methods. We have already briefly discussed different routes of synthesis for MNPs in former sections. A comparison of these methods is summarized with merits and demerits is given in Table 1, which can help researchers to select the suitable method for synthesis of MNPs. However, when a comparison is made between physical and chemical methods, the size of NPs in nanometer range is difficult to attain through physical methods (Cuenya, 2010). It is difficult to adjust the particle size and shape through the physical mode of synthesis (Decastro and Mitchell, 2002). While through chemical methods, the size and shape can be controlled by adjusting different conditions of reaction (Wu et al., 2008). Among different chemical methods, the hydrothermal method is considered as the most convenient approach to synthesize MNPs. The hydrothermal method is versatile and is superior over other methods such as sol-gel, microemulsion because of its advantages in terms of producing NPs of desirable size, shape, high crystallinity, and homogenous composition. The hydrothermal method allows controlling the morphology of synthesized particles by decreasing the chances of agglomeration and narrow size distribution (Zahid et al., 2019).

**TABLE 1:** Different synthesis methods of MNPs with respective merits and demerits.

Synthesis methods	Merits	Demerits	References
Ball milling method	Simple, widely used, produce fine powder	Contamination of product	Benjamin (1970), Sarwat (2017)
Laser evaporation	Low experimental cost, no use of chemicals, no pollutant products	High price of laser system, needs high amount of energy	Yang (2007), Jendrzej et al. (2017)
Wire explosion method	Ecologically safe, clean, and highly productive	A little contamination of product may occur	Kotov, (2003)
Coprecipitation	Simple, large quantity	Impurities, time consuming	Jiang et al. (2004)
Thermal decomposition	Controllable size, high yield	Toxic solvents	Faraji et al. (2010)
Microemulsion synthesis	Thermodynamically stable	Low yield	Vidal-Vidal et al. (2006)
Hydrothermal/Solvothermal	Good crystallinity	Needs high temp. and pressure	Wu et al. (2008)
Sol-gel method	Highly pure, good crystallinity	Longer time, toxic organic solvents	Duan et al. (2018)
Sonochemical reaction	High crystallinity, saturation magnetization, narrow size distribution	Mechanism is still not well understood	Unsoy et al. (2012)
Microwave	Fast, rapid kinetic for crystallization	Homogenous nucleation due to uniform heating	Namboodiri and Varma, (2001)
Chemical reduction	Simplicity, safe	Environmental pollution	Sun et al. (2007)
Chemical vapor deposition	Wide range production of materials	Low productivity, impurities	Wang et al. (2003)
Arc discharge	Simple, low cost	Difficult to control particle size	Faraji et al. (2010)
Laser pyrolysis	Highly localized heating and rapid cooling	Expensive	Hasany et al. (2012)
Combustion synthesis	Simple, fast, low cast	Generate impurities	Borysiuk et al. (2008)
Annealing	Controllable particle size and chemical composition	Generate impurities	Wang et al. (2007)
Biological method	Efficient, clean process, ecofriendly	Poor dispersion of NPs	Komeili, (2012)

The coprecipitation method is preferred because of its simplicity and ease in the synthesis of MNPs. The yield is high but the shape control is sometimes not that good. The sol-gel method has its advantages of high purity and crystallinity, homogeneous composition, and cost-effective because the process can be completed at room temperature. The microemulsion is suitable for the synthesis of monodisperse NPs with various morphology but of low yield. The thermal decomposition method is preferred for attaining NPs of a smaller size as compared to the coprecipitation method. Among different methods, thermal decomposition is considered the best method so far for producing NPs of controlled size and morphology (Faraji et al., 2010). Conditions like pH, types of solvent and surfactant, ionic strength, agitation, reaction time, and stirring rate are important consideration for selection of synthesis methods.

On the other hand, the biological method is an acceptable approach and is opted for its environmentally friendly, cost-effectiveness, sustainability, reproducibility, and high yield. Biological synthesis through plants is under developmental stages and researchers are still investigating to understand the mechanism (Gul et al., 2019). The NPs synthesized through microbes are not monodispersed and the synthesis process takes a lot of time as compared to chemical and physical methods (Narayanan and Sakthivel, 2010). Therefore, opinions on the selection of methods may vary from researcher to researcher based on their findings and purpose of application. That is why not a single method is referred to as the optimal method for the synthesis of MNPs. Every method has its limitation and its selection depends on many other factors like the yield of NPs, its morphology, size, shape, and experimental cost.

## Characterization of MNPs

The MNPs are characterized by different instruments to examine their physicochemical properties. The size of NPs plays a key role in the demonstration of different physicochemical properties. Even a small variation in their nanoscale dimension can change their properties. Some of the instruments used for their characterization are Atomic Force Microscopy (AFM), Energy Dispersive X-ray Diffraction (EDXD), Scanning Electron Microscopy (SEM), Fourier Transform Infrared (FT-IR) Spectroscopy, UV Spectrophotometer, Transmission Electron Microscopy (TEM), and Mossbauer Spectroscopy (MS) (Galloway et al., 2015).

### Size and Surface Morphology

Changes in the size and morphology of MNPs can change their physicochemical properties. The Brunauer Emmet Teller (BET) and Dynamic Light Scattering (DLS) techniques are used to measure surface area, size, and particle distribution. While techniques like AFM, TEM/HRTEM, and SEM/FESEM can determine the surface morphology of MNPs. The images we get through these instruments give us an idea about their shape and size from which their diameter can be calculated. The AFM technique is used to measure surface roughness, step height, and position of distributed particle. TEM is useful for the information regarding, composition, morphology, and size of NPs. While SEM gives us data regarding surface topography and composition of the samples. If the surface of the nanomaterials is nonconductive then ultrathin electrical conducting elemental sputtering is practiced. High-resolution TEM (HRTEM), field-emission SEM (FESEM), and XRD techniques can measure size calculation. TEM is very helpful in achieving the determination of the crystallinity, aggregation state of NPs, lattice spacing, and electron phase shift (Gabbasov et al., 2015; Chekli et al., 2016). The sharp peaks of XRD are easy to calculate the size of NPs which is done through the Scherrer equation. Broad peaks are obtained for non-crystalline NPs which is difficult to determine the size. XRD is used to define the crystallinity of NPs. Photon correlation spectroscopy, Mossbauer spectroscopy, and DLS techniques can measure the average size of the particle and their distribution.

### Elemental Mapping/Composition

Different instruments like EDS/EDXD, XRF (X-ray fluorescence), TEM, SEM, and XPS (X-ray photoelectron spectroscopy) can determine elemental composition and surface morphology. The instruments Inductively-Coupled Plasma Mass Spectroscopy (ICP-MS) and Atomic Absorption Spectrophotometry (AAS) are also used to determine the elemental composition in NPs. However, AAS cannot determine elemental composition when the NPs are in solid form and need to be dissolved in acids or bases before use. The XPS can provide information on composition and chemical state of elements present in a NPs. The EDXD technique is helpful for the elemental analysis and chemical composition of prepared MNPs (Faraji et al., 2010). The information about the elemental composition of a sample can be determined through XRF. The sample preparation for XRF analysis is easy, fast, and safe as compared to other methods. It can detect the amount of element up to 100 ppb (parts per billion) (Ida et al., 2005; Al-Eshaikh and Kadachi, 2016). It’s a non-destructive method of analyzing solid sample through X-radiations (Weltje and Tjallingii, 2008).

### Bonding Type and Structure

The structure and bonding characteristics of MNPs are determined with various techniques. The techniques use are FT-IR, XAS (X-ray absorption spectroscopy), TGA (thermogravimetric analysis), XPS, and RS (Raman spectroscopy). The XPS is suitable for the surface configuration of NPs, it reveals the mechanism of reactions that takes place on the surface of MNPs, that provides data on the structure and speciation of elements (Karabelli et al., 2008). FT-IR and XPS help in finding the bonding between organic and inorganic, particles binding energy, and oxidation state. FT-IR spectroscopy is also helpful to understand the functional groups of organic molecules. The RS technique is done to find out the structure and spinal lattice of the compound. TGA technique is used to estimate binding efficiency on the surface of the particle by providing us coating formation information especially about surfactants and polymers. The XAS technique is useful for information regarding oxidation states and required elements of electronic configuration (Reddy et al., 2012).

### Magnetism

The magnetic characteristics of MNPs depends on its formation based on different synthetic routes. The MNPs size ranges between nano-to-micro scales, show the property of superparamagnetic. When an external magnetic field is applied on these NPs, they show magnetic sensitivity and can interact with these external magnetic fields (Pathak et al., 2021). However, in the absence of external magnetic field shows no magnetism. This property of MNPs can make them play a vital role in targeted drug delivery and controlled therapy. The magnetism of NPs is measured with several techniques that vary in providing the quality of information and sensitivity. Techniques like VSM (vibrating sample magnetometer) and SQUID (superconducting quantum interference device) magnetometry are used to measure the net magnetization. The SQUID is useful in investigating the samples in different forms i.e., thin films, crystals, powders, liquids, and gases. Both VSM and SQUID are highly sensitive instruments e.g., VSM sensitivity is 10^–6^ emu, while SQUID is more sensitive up to 10^–10^ emu (Zahid et al., 2019). The SQUID and VSM can also determine the magnetic saturation and residual magnetization if the external magnetic field is constantly applied (Krzyminiewski et al., 2018). While the paramagnetic center and free radicles can be detected by the EPR (Electron Paramagnetic Resonance) technique. Mossbauer spectroscopy obtains data regarding bonding, structural, magnetic characters, and determination of oxidation state. The VSM can evaluate magnetizations of MNPs generally between −3 and 3 T when the external magnetic field is applied. It is also helpful in estimating the shell’s effect on the magnetization saturation (Faraji et al., 2010). Physical Property Measurement System (PPMS) is another reliable technique used to determine the magnetic properties and the magnetic behavior of NPs. This system is designed to measure magnetization vs. magnetic field and temperature of MNPs samples (Andersson, 2013; Singh et al., 2017).

## Composition of Magnetic Nanostructures

### Monocomponent Magnetic Nanostructures

#### Fe, Ni, Co-Based Magnetic Nanostructures

Iron NPs are a special kind of MNPs having unique magnetic properties. The most extensively studied NPs in the nanomedicine field because of their magnetic features and super biocompatibility. They exhibit good physical and chemical stability, less expensive, and environmentally safe. Previously, in the presence of oleic acid (OA), iron carbonyl [Fe (CO)_5_] was decomposed to form monostructure Fe NPs (Suslick et al., 1996). Keeping in mind the sensitivity of iron NPs to oxygen, iron NPs were prepared through a facile aqueous phase synthesis method which involved poly (N-vinylpyrrolidone) (PVP) useful in antioxidation of the metal surface (Hou and Gao, 2004). Monodisperse nickel (Ni) NPs were prepared through reduction of Ni (acac)_3_ in the presence of hexadecyl amine (HDA), the average size of particles was 3.7 nm (Hou and Gao, 2003). Where cobalt (Co) NPs of size ranging 2–6 nm were prepared in the presence of a bulky trialkyl phosphine reducing agent while NPs of a larger size ranging 7–11 nm were synthesized in presence of less bulky trialkyl phosphine, which shows the coordination surfactant trialkyl phosphine with neutral metal surface sites (Sun and Murray, 1999).

#### Metal Alloys Magnetic Nanostructure

Metal alloy NPs are highly promising with super magnetic featured NPs. Iron-Platinum (FePt) and Iron-Palladium (FePd) are represented examples of metal alloy nanostructure because of being high chemical stability and magnetic crystallinity (Hou et al., 2004). FePt NPs are very useful in the field of biomedicine application, synthesis through solution-phase, or vacuum deposition. FePd NPs were prepared using adamantine carboxylic acid and tributyl phosphine as stabilizers at room temperature through the organic phase thermal decomposition method. The FePd NPs show super magnetic properties with a tunable size of 11–16 nm. Previously, wet chemical approach was used to prepare monodisperse FePd NPs of fcc (face centered cubic) shape by reducing Pt (acac)_2_ and decomposing Fe (CO)_5_ (Sun et al., 2000). But on conversion to fct (face centered tetragonal), it tends to aggregate. By thermal annealing, the fcc-Fe_3_O_4_ NPs with Mg coating was converted to fct-FePt NPs (Liu et al., 2014b). The layer of magnesium oxide (MgO) prevented NPs from aggregation. FePt NPs with gold coating were synthesized by reducing Pt (acac)_2_ and decomposing Fe (CO)_5_ in octyl ether solvent. The gold shell around these NPs made them highly biocompatible (De La Presa et al., 2007). Some other binary metallic alloys of high magnetic properties comprising of iron and cobalt also exist (Fe_12_Co_88_, Fe_40_Co_60_, Fe_60_Co_40_), their oxidation is prevented by coating with gold, silver, or graphitic (Bai and Wang, 2005; Seo et al., 2006).

#### Metal Oxide Magnetic Nanostructures

Metal oxide NPs recently got high attention due to the magnetic features and possessing chemical stability. One of them is Fe_3_O_4_, which has shown a promising potential application in biomedicine and magnetic separation. They are prepared by a simple process mainly based on complexes of Fe under alkaline conditions, (Hou et al., 2003). The desired size can be obtained by controlling the solvent and surfactant (Sun and Zeng, 2002; Xu et al., 2009). Oleyl amine, a surfactant, was contacted with Fe^3+^ to make Fe^3+^ −OOC−, particle growth was restricted during this process because of acting as capping agent. Different shapes of iron oxides like octahedral Fe_3_O_4_ NPs and Fe_3_O_4_ nano prisms can be obtained when oleyl amine surfactant is used for tuning the energy of particle surface to facilitate growth in a specific direction (Zhang et al., 2009; Zeng et al., 2010). The amount of surfactant used can control particle growth in a particular direction (Hou et al., 2007b). Moreover, NPs of FeO can also be transformed into iron oxides (Fe_3_O_4_) after thermal annealing. Similar routes were followed for making cobalt oxides. During a hydrothermal process, cobalt nitrate [Co (NO_3_)_3_] formed nanoplatelets (NPLs) of cobalt(II) hydroxide [Co(OH)_2_] in PVP presence, which may further result in cobalt oxide (CoO) NPLs (Hou et al., 2005).

#### Metal Carbides Magnetic Nanostructures

Despite the remarkable magnetic properties and stability, iron carbides (Fe_5_C_2_, Fe_3_C, and Fe_2_C). They are rarely studied because of the challenges faced in their synthesis associated with controlling the size and morphology. NPs of Fe_5_C_2_ were produced by decomposing Fe (CO)_5_ in presence of octadecyl amine (Yang et al., 2012). Iron NPs of very good crystalline structure were carbonized to synthesize Fe_5_C_2_. For tuning the surface energy, Bromide was added during the synthesis of carbide NPs, but it was unclear to understand the mechanism. The Fe_5_C_2_ NPs of 20 nm size produced through this way had an amorphous shell. A synthetic chemical route was developed in which iron carbide NPs with different structures of crystallinity were formed (Yang et al., 2017). These iron carbides exhibit weak ferromagnetic properties, which depicted that the synthetic routes followed for the formation of these iron carbides may result in different kinds. Especially when halide ions are introduced, could be responsible for affecting the penetration of carbon content through selective absorption.

### Multicomponent Magnetic Nanostructures

#### Heterostructure Magnetic Nanostructures

The MNPs with heterostructures are of a diverse composition which comprises a magnetic part with some other components, together they exhibit special properties. These different components integrated to become a multicomponent magnetic structure provide newfangled properties. Monocomponent were used as seeds to prepare MNPs through a seed-mediated process. Investigations are being carried out to study core@shell heterostructures which consist of different components enclosed in shells that exhibit synergistic effects. For example, NPs of Fe_3_O_4_@Au@Ag were synthesized with controllable properties (Xu et al., 2007). FePt based heterostructure like FePt-Au was widely studied for showing magnificent magnetism. Au is famous for its wide applications in the field of biomedicine or catalysis. FePt-Au heterostructure nanowires (HNWs) were synthesized in which Au NPs growth over FePt NRs was observed (Wu et al., 2011). Tunable structures of FePt-Au can be prepared by controlling Au complexes. A similar method was followed to prepare FePt-Au heterostructure NPs were developed. (Zhu et al., 2013).

#### Exchange Couple Magnetic Nanostructures

The effect of an exchange-coupled between a hard and soft origin magnetic phase is of great importance to meet the requirements of a higher energy barrier product. When particles with a soft origin magnetic phase are smaller in size than the particles with a hard magnetic phase, it will behave like a single phase. Theoretical calculation indicated that if the size is smaller than twice the thickness of the hard-magnetic phase may have an effective exchange coupling effect. But this can be done under very controlled conditions during synthesis. The chemical synthesis approach provides a complete understanding of developing the magnetic moment at the nanoscale (Skomski and Coey, 1994; Liu et al., 2014a). An effective method to create an exchange coupling effect on hard magnetic cores is coating with soft magnetic shells, which can increase their magnetization (Liu et al., 2014b). The seed-mediated synthesis is effective to produce high-performance exchange-coupled nanomagnets. The exchange-coupled nanocomposites of Nd_2_Fe_14_B-based were investigated to increase (BH) max (Yu et al., 2014). In similar way nanocomposites of SmCo_5_-based were also prepared (Hou et al., 2007a). As discussed earlier, for obtaining mono and multicomponent MNPs the most effective approach is chemical synthesis. The MNPs of the desired size depend on the conditions we set for chemical reactions during chemical synthesis. Different parameters are set for obtaining the desired type of MNPs with intrinsic properties. The chemical synthesis of MNPs offers different approaches to produce NPs with superparamagnetic features.

## Application of MNPs

The MNPs in the past decade have gained great attention because of their promising results in various fields. MNPs with super-magnetic properties, unique size, shape, high surface area and volume ration, and biocompatibility make their application more promising. Due to these properties, it has attracted more researchers from different fields. In this review, we have summarized the applications of MNPs in some well-known fields such as biomedicine, biosensing, environment, agriculture, and catalysis. A brief description of MNPs potential applications in these fields are provided below.

### Biomedicine

The MNPs are recently widely used in many biological applications due to their diverse physicochemical properties, easy preparation, stability, and biocompatibility. MNPs can interact with external magnetic fields. MNPs also can alter the magnetic fields in their vicinity, hence elevate magnetic resonance imaging (MRI). The externally applied magnetic field produce and generate different type of force and torque at dipoles which results in the translation, rotation, and dissipation of energy. Such type of phenomena shows plenty of applications in cell separation/biomarker, delivery of targeted drug magnetically, actuation of cell surface receptors via magneto-mechanical, biomedical imaging, bacterial theranostics, triggering of drug release, and hyperthermia. The formulation of MNPs composed of several materials showing varying physical and magnetic characteristics depending upon their usage in many applications. However, in biomedical research the most important factor one must consider their potential biocompatibility/toxicity (Lewinski et al., 2008; Kong et al., 2011).

#### Cancer Theranostics

In the last few decades, nanobiotechnology and molecular biology fields emerged as novel approaches for cancer theranostics (Nam et al., 2019; Gong et al., 2020). These systems mainly involve the design and fabrication of engineered NPs with multifunctional properties that address the limitations of conventional cancer diagnostics and therapeutic agents (Chen et al., 2017; Overchuk and Zheng, 2018). NPs can be used to construct nanoscale imaging probes for early detection and visualization of cancer development (Goel et al., 2017). In addition, NPs are also being designed as vehicles to effectively deliver anticancer agents, genes, or proteins to the targeted tumor sites *via* enhanced infiltration and retention (EPR: Electron Paramagnetic Resonance) effect (Chen et al., 2017; Overchuk and Zheng, 2018; Zhao et al., 2020).

Although promising, however, there is still an urgent need to develop novel nanomaterials that can meet the requirements of emerging cancer theranostics applications. In this context, MNPs offer unique physicochemical properties and super magnetic characteristics, which make them ideal candidates for hyperthermia therapy cancer (Liu et al., 2020), MRI (Farzin et al., 2020), biosensing (Kang et al., 2017), and targeted drug delivery (Han et al., 2020).

**FIGURE 2:**
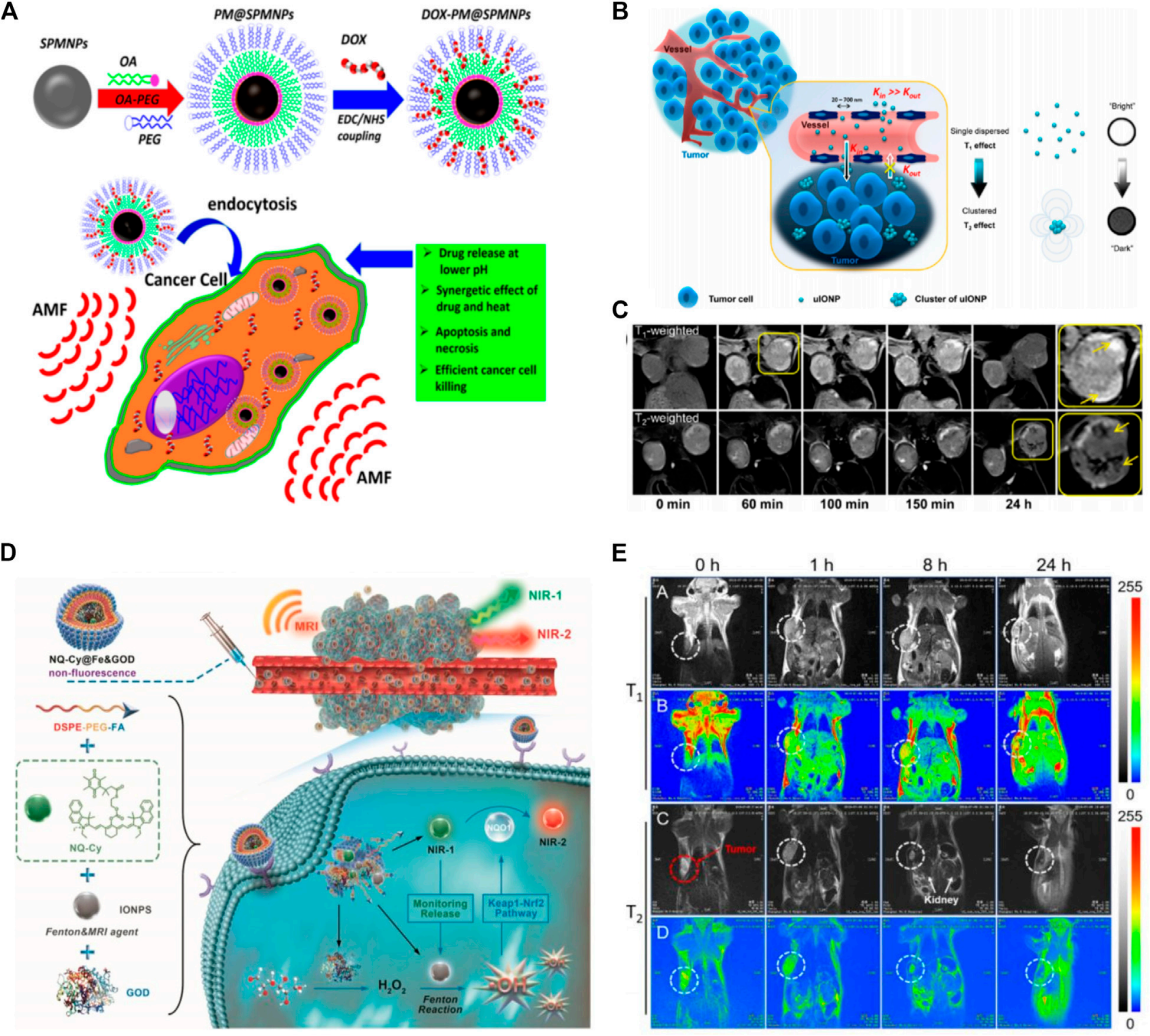
Schematics **(A)** illustration of the anticancer drug (DOX) conjugating into polymer encapsulated superparamagnetic NPs and the overall mechanism of cancer cell killing by synergistic therapy under applied Alternating Magnetic Field (AMF). Copyright 2017, American chemical Society (Thorat et al., 2017) **(B)** Representation of the mechanism of improved EPR effect and accumulation of ultrafine iron oxide nanoparticles (uIONPs) inside tumor with a bright-to-dark T_1_-T_2_ MRI contrast transition. uIONPs quickly and easily extravasate from dripping tumor vessels into a tumor with favorable kinetics, then self-assemble into clusters in the tumor microenvironment at a low pH (6.5), limiting clustered uIONP intravasation back into circulation **(C)** Optical images of T_1_-and T_2_-weighted MRI of a mouse with orthotopic 4T1 tumors pre- and post i.v administration of uIONP at different time intervals (Wang L. et al., 2017). Copyright 2017, American Chemical Society **(D)** Tumor-specific nanotheranostics NQ-Cy@Fe&GOD for *in situ* real-time reporting of Fenton-based dose-dependent •OH generation: 1) Owing to the passive and active targeting ability, NQ-Cy@Fe&GOD are delivered into tumor sites, along with the enhanced MRI signal; 2) Turn-on NIR fluorescence signal at 830 nm (NIR-1) is observed in synchronous with its intracellular dissociation for evaluating the released dose of Fenton agents. Concurrently, the released GOD catalyzes glucose to produce H_2_O_2_; 3) Subsequently, high-level •OH is generated by IONPs-based Fenton reaction with intratumor H_2_O_2_, thereby triggering the activation of Keap1-Nrf2 pathway for high-level overexpression of NQO1 enzyme, which is timely supervised by a new emission at 650 nm (NIR-2). Overall, this nanocomposite bridges dual-channel NIR imaging and MRI modality, thereby uniting spatial and temporal resolution on dose-dependent •OH therapeutic feedback **(E)** Imaging of the bio-distribution with MRI signal by intravenous injection. T_1_-Weighted **(A,B)** and T_2_-Weighted **(C,D)** MRI of tumor-bearing mice after experiencing a magnetic field at after the intravenous administration of NQ-Cy@Fe&GOD at different time intervals. Copyright 2020, John Wiley and Sons (Ma et al., 2020).

Hyperthermia treatment is an irreversible thermal ablation of pathologic targets by raising the localized temperature of tissue through the induction of heat. Magnetic hyperthermia is a new treatment in cancer therapies, where an external Alternating Magnetic Field (AMF) is applied to generate heat in suspensions of MNPs within the body (Noh et al., 2017; Pathak et al., 2017; Pathak et al., 2019; Liu et al., 2020). Cancerous cells are more vulnerable to hyperthermia than normal cells since the pH in the cancerous microenvironment is lower, resulting in reduced thermotolerance (Li et al., 2020). This strategy is considered a promising approach for cancer treatment with specific localized hysteric heat and minimal heating of background tissues. The most important advantage of MNPs-based hyperthermia therapy is its deep tissue penetration and magnetism-assisted specific killing of cancer cells without damaging healthy tissues (Yoo et al., 2011; Moros et al., 2019). The MNPs-based hyperthermia aids in the realization of intracellular hyperthermia, as it provides therapeutic heating directly to cancer cells (Del Sol-Fernández et al., 2019). The intracellular hyperthermia modality can be further enhanced by coupling cell-targeting ligands to MNPs for selective targeting. This localized and selective heat greatly improves the efficacy of cancer treatment (Sharma et al., 2019). Owing to these advantages, MNPs-based hyperthermia treatment for tumor eradication has recently come out from the lab to clinical trials (Espinosa et al., 2018; Moros et al., 2019). The MNPs not only can be used as vehicles for delivery and controlled release of drug upon exposure to the external field but can also be used to enhance chemotherapy through magnetic field-mediated hyperthermia. Previously, a polymeric micelle structure was designed by functionalizing superparamagnetic La0.7Sr0.3MnO_3_ nanoparticles (SPMNPs) with an oleic acid-polyethylene glycol (PEG) with a high loading capacity for anticancer cancer drug doxorubicin (DOX) to deliver into cancer cells (Thorat et al., 2017). Authors reported that SPMNPs not only enhanced the drug delivery but also increased cancer cells by magnetic hyperthermia-based synergistic mechanism. In addition, SPMNPs loaded with drugs improved the chemotherapeutic effect upon triggering with external AMF. Moreover, both heat generation and drug release can be monitored for on-demand synergistic Hyperthermia/chemotherapy by simply adjusting the AMF frequency (Figure 2A).

Medical imaging is regularly used to investigate biological phenomena, diagnose abnormalities, and monitor disease progression. To enhance the resolution of medical images, novel imaging techniques are being developed. Several imaging techniques including magnetic resonance imaging (MRI), computed tomography (CT), positron emission tomography (PET), single-photon emission computed tomography (SPECT), ultrasound (US) imaging, optical fluorescence imaging, and photoacoustic (PA) imaging, are currently in practice for anatomical diagnosis. In comparison to CT and PET, MRI is an appealing imaging approach and an effective cancer diagnosis tool with excellent capabilities such as high spatial resolution, less radiation exposure, and high contrast imaging of soft tissues (Schenkman, 2011; Kircher and Willmann, 2012). Nuclear magnetic resonance (NMR) and MRI are based on the same concepts, however, in MRI a strong magnetic field is applied to a body or specimen to gain a lower or higher energy state. In MRI T_1_/T_2_ images describe a bright-to-dark contrasting signal, where T_1_ represents recovered and T_2_ represents residual magnetization after specific time intervals. The size of the MNPs is considered an important parameter for MRI imaging. In magnetic cancer theranostics, ultrafine iron oxide NPs allows to monitor the position of theranostics agents, manage the therapeutic process in real-time, and assess treatment efficacy (Wang L. et al., 2017). The ultrafine iron oxide NPs as dual MRI contrasting agents (T_1_/T_2_ contrast switch), could penetrate the deep-seated tumor and provide multimodal imaging modalities (Figure 2B). Moreover, the NPs self-assembled or formed a cluster in an acidic tumor environment and prevented re-entry into circulation, retained inside the tumor for a long-time, thus enhancing imaging resolution in MRI signaling (Figure 2C).

In recent decades, a lot of progress has been made in cancer immunotherapy. In immunotherapies, cancer vaccines, particularly immunoadjuvant agents are considered emerging approaches for tumor elimination and have sparked intense research interest. Recently, the employment of electromagnetic-based nanomedicines to enhance cancer immunotherapy has attained great research attention. A variety of MNPs has been explored to generate heat/reactive oxygen species upon activation by electromagnetic energies. These electromagnetic energy-triggered nanomaterials have been widely used for photothermal therapy, photodynamic therapy, sonodynamic therapy, or radiotherapy, and magnetic hyperthermia to cause immunogenic cell death. In photothermal thermal-based immunotherapy immunoadjuvants such as cytosine-phosphate-guanine (CpG) oligodeoxynucleotides (ODNs) and resiquimod (R848) have been studied to potentiate immune response. Previously, magnetic-responsive immunostimulatory nano agents (MINPs) loaded with superparamagnetic iron oxide NPs (SPIO-NPs) and CpG-ODNs were used to develop a photothermally activated immunotherapeutic nanosystem (Guo et al., 2019). The clinically approved components in the designed system served as a magnetic-targeting therapeutic agent for both photothermally stimulated immunotherapy as well as a contrast agent for photoacoustic (PA)/magnetic resonance (MR) bimodal imaging. Upon exposure to Near infrared (NIR), the MINPs provided an efficient photothermal conversion effect to generate heat-effective photothermal ablation of primary tumors, releasing immunological agents, and activated immune response.

Reactive oxygen species (ROS) are chemically reactive molecules that play important functions in cell survival at lower concentrations, but toxic to a cell at a certain threshold leading to cell apoptosis or necrosis (Ramsey and Sharpless, 2006). Hydroxyl radical (•OH) is the most effective oxidant among the reactive oxygen species (ROS), and it plays a critical role in ROS-mediated cancer cell death and tissue damage (Yang et al., 2019). In recent years, ROS-mediated cancer treatment based on *in situ* ROS generation has gained widespread research interest (Trachootham et al., 2009). Because of the short lifecycle and high reactivity of •OH, the *in-vivo* therapeutic response of •OH efficiency is typically impulsive, making the therapeutic ability of •OH a major bottleneck in chemo-dynamic therapy. The Fenton reaction is a typically old reaction that specifically generates •OH radical upon the disproportionation of hydrogen peroxide (H_2_O_2_) with Fe^2+^ ions (Fenton, 1894). In the Fenton reaction, Fe^2+^ ions are essential for ROS generation. However, due to their broad biodistribution across the body, after injection few free Fe^2+^ ions could reach the tumor site for ROS development. To this end, Zhu’s group designed a novel theranostics system (NQ-Cy@Fe&GOD) by encapsulating dual-channel fluorescence probe NQ-Cy, Fenton-based iron oxide NPs, and glucose oxidase enzyme with the assembly of amphilic copolymer (Ma et al., 2020). After endocytosis, the NQ-Cy@Fe&GOD system successfully regulated the intracellular release of iron oxide NPs and glucose oxidase (GOD). The GOD initiated the oxidation reaction to generate H_2_O_2_ *in situ*, and iron oxide NPs catalyzed it into •OH radical *via* Fenton mechanism (Figure 2D). Their findings revealed that MRI signal can trace the quantity of distributed Fenton-based iron oxide NPs with high spatial resolution in MRI, while the fluorescence signal quantifies the •OH-mediated therapeutic feedback with high spatio-temporal resolution (Figures 2D,E). Owing to ease in functionalization, biosafety, and particularly magnetic properties MNPs have many advantages in biomedicine, including diagnostic imaging, cancer therapy, and drug delivery, etc. In this section, we highlighted the key applications of MNPs in cancer theranostics (diagnosis and therapies) with representative examples.

#### Bacterial Theranostics

Antimicrobial resistance has posed serious health concerns in both developing and developed countries. Due to the emergence of various kinds of multidrug-resistant strains and the unavailability of new antibiotics, it is estimated bacterial infections will cause 300 million deaths by the end of 2050 (Shi et al., 2019). Such scenario has increased the demand to explore new innovative strategies in antimicrobial therapies (Kruijshaar et al., 2008; Arias and Murray, 2009; Harms et al., 2016). Up to date, several innovative approaches that are currently under investigation are antimicrobial peptides, therapeutic antibodies, phage therapy, and antimicrobial NPs (Simoes et al., 2017). Recently, MNPs based strategies have been developed to treat infections caused by multi-drug resistant bacteria as well as bacteria-related biofilm (Reddy et al., 2012; Bohara and Pawar, 2015; Zazo et al., 2016; Häffner and Malmsten, 2017; Majid et al., 2018). The MNPs kill microbes mainly through three basic mechanisms including disrupting plasma membrane, releasing toxic metals, and ROS generation that interfere major bacteria components (Reddy et al., 2012; Huang et al., 2014). Iron oxide NPs as the prominent type of MNPs have some key importance due their magnetic, hyperthermal, and catalytic properties (Prucek et al., 2011; Nosrati et al., 2017).

**FIGURE 3:**
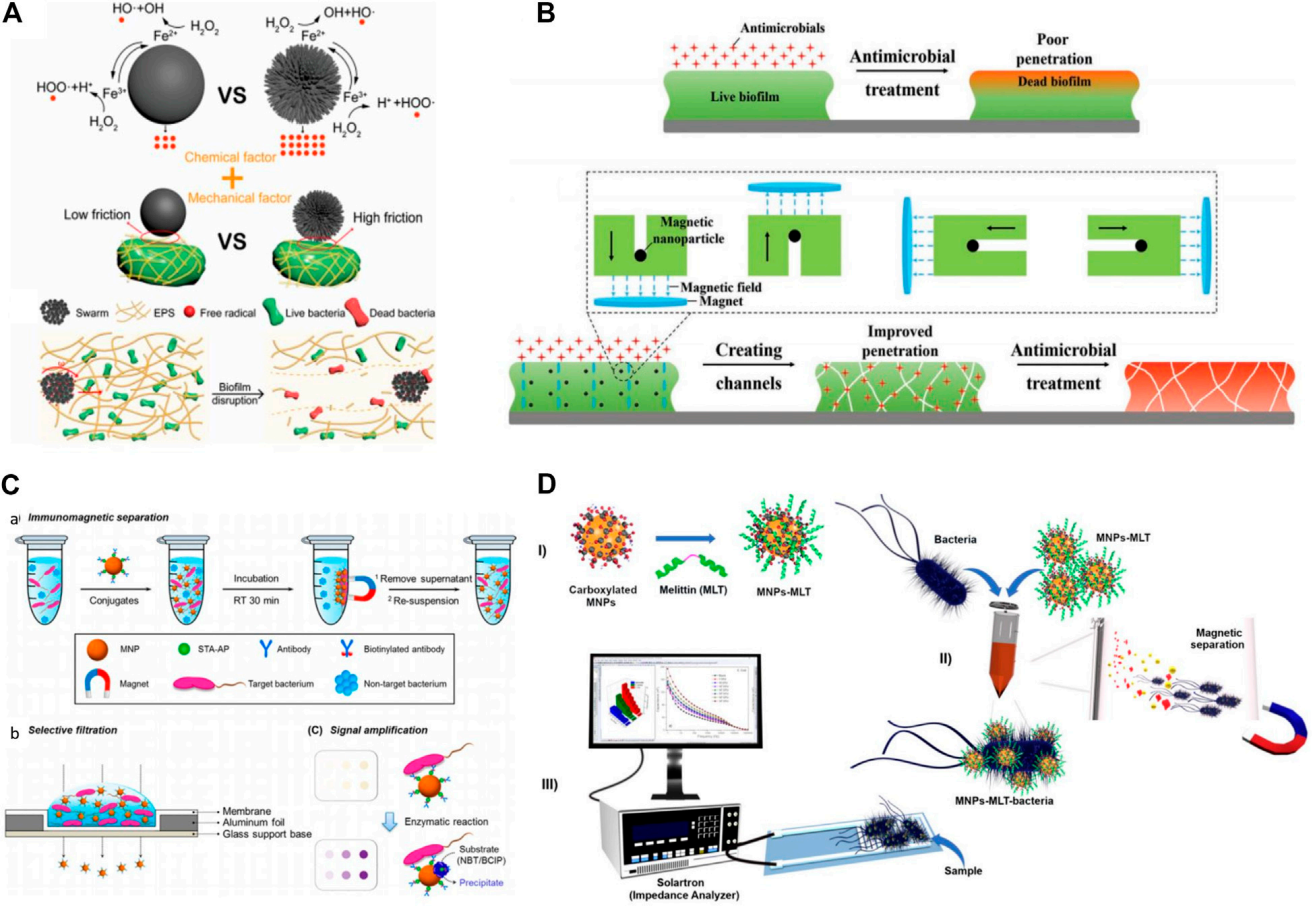
Schematics **(A)** illustration of the chemical and mechanical factors of the bacteria killing of Fe_3_O_4_ and p-Fe_3_O_4_ MPs. and mechanism of biofilm disruption by a p-Fe_3_O_4_ swarm (Dong et al., 2021). Copyright 2021, American Chemical Society **(B)** mechanistic Representation of artificial channel created by magnetic nanoparticles in infectious biofilms to improve antimicrobial penetration and enhance bacterial killing over the depth of a biofilm (Quan et al., 2019b). Copyright 2019, John Wiley and Sons **(C)** Enhanced colorimetric method using enzymatic amplification with NBT/BCIP precipitation based on IMS-selective filtration for the ultrasensitive detection of *E. coli* O157:H7: a. IMS for target bacteria using biotinylated antibody/STA−AP/MNP conjugates, b. selective filtration of target bacteria−conjugates complexes, and c. colored spots on the filter membrane by target bacteria-bound biotinylated antibody/STA−AP/MNP conjugates (upper panel) and enhanced colorimetric spots by enzymatic amplification with NBT/BCIP precipitation on the bacteria-bound biotinylated antibody/STA−AP/MNP conjugate surfaces (lower panel) (Kim et al., 2018). Copyright 2018, American Chemical Society **(D)** Representation of the principle of the bioanalytical method. Functionalization of MNPs (I); capture of bacteria by MNPs-MLT and magnetic separation of the bacteria from the sample matrix (II); EIS detection (III) (Wilson et al., 2019). Copyright 2019, Elsevier publishing group.

Biofilms are bacterial communities that adhere to surfaces and are embedded in a self-released matrix of extracellular polymeric substances (EPS) (Arciola et al., 2018). Because of the high mechanical stability and antimicrobial blockade created by extracellular polymeric substances (EPS), biofilm is hard to treat with traditional antibiotics (Koo et al., 2017). The EPS, which serve as local barriers, shield bacteria cells from the host immune system and prevent drug diffusion, thereby resulting in high bacterial resistance to antibiotics and thus making biofilm removal a challenging task (Boudarel et al., 2018). Currently, micro-/nanorobots-based drug delivery has attracted widespread interest due to their controlled and regulated motion ability for targeted cargo release. Inspired by the swarming phenomenon in biology, micro/nanorobots can move in organized collective patterns to pathologic targets (Yan et al., 2017; Schmidt et al., 2020). Recently, scientists have introduced the magnetic-field-controlled swarming of micro/nanorobots, which has proved the ability of high loading capacity and strong convections in swarm-like motion (Yu et al., 2019; Wang et al., 2021). In this pursuit, Fe_3_O_4_ mesoparticles (Fe_3_O_4_ MPs) due to stable para-magnetism are widely employed for designing of magnetic microrobots (Dong et al., 2021). In addition, they exhibit intrinsic peroxidase-like properties, which means that the Fe element in Fe_3_O_4_ MPs can proceed the Fenton reaction, generating hydroxyl free radicals (•OH) that can destroy the biofilm matrix and kill bacteria cells (Figure 3A). The underlying mechanism mainly involves two phenomena. First, the toxic bactericidal free radicals degraded biofilm matrix and killed bacteria. Second, physical disruption of the biofilm and promoting deep diffusion of •OH into biofilm by the swarming movement.

In a bacterial biofilm, water-filled channels and openings exist to facilitate the efficient diffusion of nutrients, autoinducers, and waste products (Wilking et al., 2013). For example, flagella-driven movement enables motile *bacillus* swimmers to dig water-filled channels. Artificial channels in pathogenic *Staphylococcus aureus* biofilms have been created by irrigating them with *bacillus* swimmers, making these infectious biofilms hundred times more vulnerable to benzalkonium chloride (Houry et al., 2012). Addressing the phenomenon of natural *bacillus* swimmers to dig channels in biofilms, magnetic iron oxide NPs were applied to artificially create channels in a biofilm to increase the diffusion and promote bacteria-killing by antimicrobials. The main role of magnetic iron oxide NPs was to establish artificial channels to promote the penetration of the drug into biofilm, which significantly enhanced the killing of pathogenic bacteria (Figure 3B) (Quan et al., 2019a).

The majority of pathogenic bacteria are mesophilic and can thrive best at a temperature ranging 33–41 C (Mackowiak, 1981). The elevated temperature hinders bacterial proliferation and movement, which promote increased autolysis and cell wall disruption (Tsuchido et al., 1985). Currently, Near Infrared (NIR)-triggered photothermal therapy (PTT) is considered a promising and effective antibacterial strategy due to its minimal invasiveness, deep tissue penetration, easy handling, and lack of resistance (Gao et al., 2018). Owing to efficient light absorption capability, photo-responsive nanomaterials can transform light energy into heat, which can kill bacteria by disrupting cell membranes and causing protein denaturation, which leads to cell death (Huang et al., 2019; Mao et al., 2019). Recently, multimodal photothermal therapy is recognized as an intriguing approach due to short irradiation time, reduced antibacterial dose, and improved antibacterial performance. Hemoglobin-functionalized copper ferrite nano-particles (Hb-CFNPs) was designed with integration of combined catalytic and photothermal property to synergistically eradicate pathogenic bacteria (Liu et al., 2019). In addition, the intrinsic magnetic property of Hb-CFNPs increased about 20 times photothermal efficiency through magnetic hyperthermia *via* magnetic enrichment, which enhanced the bactericidal efficacy even at a lower dose. Initially, the generated •OH caused oxidative damage to the cell membrane, increased membrane permeability, and sensitivity to heat. When combined with PTT, the damaged membrane was quickly destroyed, shorten treatment time and minimized adverse side effects of PTT to healthy tissues.

In clinics, the traditional diagnostic method used for distinguishing pathogenic bacteria often involves the cultivation of bacteria, which is recognized as the standard diagnostic technique (Pazos-Perez et al., 2016; Xu et al., 2018). However, the conventional diagnostic techniques often involve time-consuming procedures, which need professional trainers and, in some cases, demand expensive equipment (Gal-Mor et al., 2014). Therefore, prolong testing process and delaying response to patients, often affect the prescription of treatment. To address these limitations, several advanced techniques such as Enzyme-linked Immunoassay (ELISA), Polymerase Chain Reaction (PCR), Western blotting, and whole-genome sequencing, have been developed to reduce detection time and obtain more precise knowledge about the bacteria (Váradi et al., 2017). The shortcomings of these modern techniques are they not only require specific and costly instruments but also very laborious which restrain their practical use in clinics. Owing to ease in application of magnetic fields to remotely control the location of MNPs upon modification with bacteria targeting molecules has been widely employed for bacterial enrichment, discrimination, and separation. The MNPs aided in the improvement of different sensing techniques such as PCR, fluorescent detection, colorimetric detection, and surface-enhanced Raman detection to make them promising platforms for bacterial detection (Yuan et al., 2018; Xu et al., 2019). Colorimetric detection is a technique, which involves the qualitative analysis of variations in color induced by bacteria through the naked eye. Detection of bacteria by the colorimetric method has been widely improved by the integration of MNPs. For example, MNPs conjugated with bacteria-specific antibody was used to concentrate and separate bacteria by applying an external magnetic field (Kim et al., 2018). The MNPs attached or unattached to bacteria can be easily separated by vacuum pressure followed by filtration through a membrane. Hence the variation in color signals produced by residual MNPs determined the quantity of bacteria (Figure 3C). The detection of foodborne pathogens often requires simple and rapid techniques beyond the standard methods which are not comply with routine analysis in food technology. Currently, biosensors comprised of impedance spectroscopy equipped with antimicrobial peptides functionalized interdigitated electrodes (IDEs) have been applied to detect pathogens (Etayash et al., 2014). However, the use of AMPs in such biosensors often suffer due to low selectivity of these molecules and less activity against bacteria, fungi and viruses (Zasloff, 2002). To this end, an electrical impedance spectroscopy-based biosensor was developed with the integration of AMP (melittin) functionalized MNPs coated screen-printed digitated electrode to detect bacteria in food samples (Figure 3D) (Wilson et al., 2019). The analysis reveals that such sensitive biosensor can detect bacteria at a very low colony forming units (CFUs). The combination of MNPs and AMPs can allow to design a highly sensitive, fast, and cheaper bioassay for detection of bacteria in potable food samples. In this section, we summarized the significance of MNPs-based materials for the treatment and diagnosis of pathogenic bacteria with representative examples.

#### Biosensing

**FIGURE 4:**
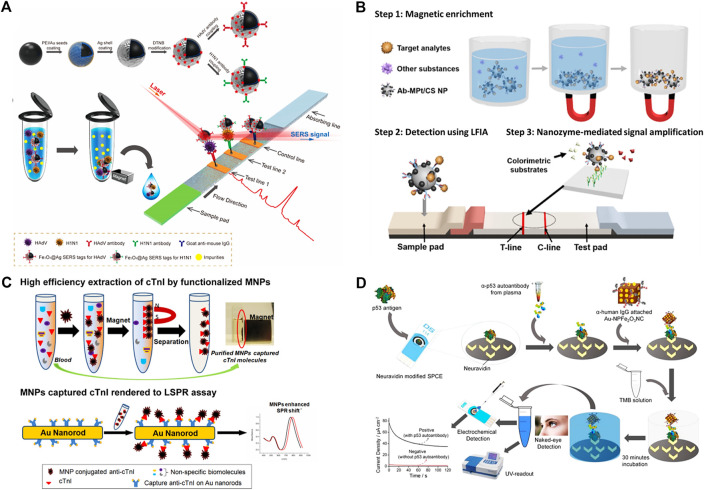
Schematics of **(A)** Synthetic Route for Antibody-Modified Fe_3_O_4_@Ag Magnetic Tags and diagram of the Magnetic SERS Strip for Detecting Two Respiratory Viruses (Wang et al., 2019). Copyright 2019, American Chemical Society **(B)** Analyte Detection Using Magnetic Nanozyme-Based LFIA Strips (Kim et al., 2017b). Copyright 2019, American Chemical Society **(C)** Bio-separation of target molecules from blood plasma by functional Fe_3_O_4_ magnetic nanoparticles (MNPs), followed by the MNP mediated nano-SPR assay. The application of MNP results in an enhancement of the LSPR shift at peak absorption wavelength (Tang et al., 2013). Copyright 2017, American Chemical Society **(D)** Assay for the detection of tumor-associated plasma (and serum) p53 autoantibody. A neutravidin-modified screen-printed carbon electrode was functionalized with biotinylated p53. Serum/plasma samples containing p53-specific autoantibody were then incubated onto the electrode surface followed by the incubation with IgG/Au−NPFe_2_O_3_NC nanocatalyst. The surface-attached Au−NPFe_2_O_3_NC nanocatalyst catalyzed the oxidation of TMB in the presence of H_2_O_2_ and produced a blue-colored complex product (naked eye), which turned yellow after the addition of an acid to the reaction media. The level of p53 autoantibody was detected *via* measuring the intensity (UV−vis) and amperometric current generated by the yellow product (Masud et al., 2017b). Copyright 2017, American Chemical Society.

The MNPs-based sensors have shown remarkable application in different fields including food technology, lab-testing, clinical diagnosis, and environmental monitoring (Haun et al., 2010; Rocha-Santos, 2014). Particularly, owing to biocompatibility, durability, and safety, the MNPs-based biosensing industry has attained good interest in the field of nanomedicines (Lin et al., 2017; Wang et al., 2017b). Due to small size, high sensitivity, and interesting noninvasive detection property, MNPs-based biosensors have envisioned a wide application in the biomedical field (Chen et al., 2016). In comparison to other conventional biosensors, MNPs-based biosensors have achieved tremendous turned out due to distinct properties as magnetic signaling and magnetic separation. Moreover, because of the high signal-to-background ratio MNPs can be used as magnetic probes to detect the analytes in biological samples (Xie et al., 2011). The MNPs are made up of magnetic-origin elements including iron, cobalt, and nickel, as well as their oxides (Mornet et al., 2006). Since superparamagnetic particles can only be magnetized by an external magnetic field, hence there is no potential magnetization in the absence of an external magnetic field (Majetich and Jin, 1999). Early and accurate detection of respiratory viruses is critical for preventing infection and directing possible treatment (Vandenberg et al., 2020). In biosensing, lateral flow immunoassay (LFIA) strips due to easy handling, low cost, and short assay time has gained tremendous interest in point-of-care testing (POCT) technology for various applications (Huang et al., 2016). For example, a study reported a Fe_3_O_4_@Ag magnetic tags-based SER-based strip (SER: Surface-Enhanced Raman Scattering) for detecting two respiratory viruses (Wang et al., 2019). The Fe_3_O_4_@Ag magnetic tags were conjugated with double-layer Raman dye and virus-sticking antibodies with abundant magnetism to specifically target viruses in a solution and SERS detecting signals of viruses on the strip (Figure 4A). The magnetic SERS strip can be employed directly for real biological samples without any sample pretreatment measures. The system has shown very low detection limits for both viruses, while 2000 times more sensitive than the standard colloidal gold strip. Similarly, in other study, Pt-decorated magnetic nanozymes based bioassay was developed with unique properties and high sensitivity (Kim et al., 2017a). Ferromagnetic Fe_3_O_4_ NP is a well-known nanozyme with superior catalytic activity over natural enzymes. However, the incorporation of Pt to the outer spaces of Fe_3_O_4_ NPs built a hybrid nanostructure (MPt/CS NPs) with improved catalytic activity (Figure 4B). The magnetic properties allowed for the magnetic enhancement of liquid samples, while their catalytic properties enabled for signal amplification through enzyme-mimic reactions.

The efficient separation of biomolecules is mainly dependent on the diverse functional groups and a high saturation magnetization value (Fatima and Kim, 2017). Magnetic-based microspheres or composites include a large number of magnetic bodies, porous polymeric structures, and specific metalcore, which offer higher affinities for targeted biomolecules (Fu et al., 2009; Haun et al., 2010). The localized surface plasmon resonance (LSPR) of metal NPs can be greatly enhanced by ferromagnetic NPs. These NPs possess a high refractive index and molecular weight make them effective candidates for enhancing the plasmonic response to biological binding events, thereby enabling the detection of small molecules even in trace amounts (Figure 4C) (Tang et al., 2013).

Recently, nanozymes with peroxidase-like activity have been employed to detect analytes in biological samples (Luo et al., 2010). In this pursuit, peroxidase mimics gold-loaded nano-porous ferric oxide nano-cubes were used for naked-eye detection of p53 autoantibodies with electrocatalytic and colorimetric mechanism (Masud et al., 2017a). A biosensor was designed by immobilizing biotinylated p53 antigen on a neutravidin-modified screen-printed carbon electrode (SPCE), and then serum or plasma samples containing the target antibody were added. The electrode surface was then coated with human IgG-functionalized Au–NPFe_2_O_3_NCs, which formed an immunocomplex with the target p53 autoantibody (Figure 4D). To promote the nanocube-catalyzed oxidation of TMB, the electrode surface was coated and incubated with a freshly prepared TMB/H_2_O_2_ solution. Colorimetry and chronoamperometry were used to observe and quantify the color transition. MNPs can also be effectively functionalized with a variety of biochemical substances for detecting protein, enzymes, nucleic acid, and cells (Haun et al., 2010; Tran et al., 2010; Suaifan et al., 2013; Zhang et al., 2013). The interesting physicochemical properties and stability of MNPs make them effective candidates for detection in both *in vitro* and *in vivo* without disturbing biological interactions. Many extensive efforts have been made to design MNPs biosensors with easy operation and high sensitivity for accurate detection.

### Environment

The deterioration and contamination of water, soil, and atmosphere are becoming a foremost environmental problem due to the increased release of toxic and lethal chemicals and compounds as a result of anthropogenic activities. Various kinds of organic pollutants like polychlorinated biphenyls (PCBs), polycyclic aromatic hydrocarbons (PAHs), pharmaceutical, pesticides, and industrial wastes are persistently present in the atmosphere (Jones and De Voogt, 1999; Rodriguez-Narvaez et al., 2017; Richardson and Ternes, 2018). Several kinds of organic pollutants are present in drinking water, sewage effluents, seawater, and groundwater. Such kind of persistent organic pollutants may pose serious health problems to the human when it becomes part of the food chain (Jin et al., 2014; Govan, 2020). To make the quality of water better it’s very important to design and develop efficient technologies. Recently, nanotechnology is one of the beneficial and more reliable options over conventional treatments. The nanomaterials, carbon, and metal oxide have been reported for the treatment of water and air (greenhouse gases, adsorption of bioaerosols, thermal decomposition, and the catalytic degradation of pollutants). The direct injection of iron NPs in the subsurface under pressurized conditions has proved to degrade the chlorinated compound i.e., trichloroethylene, to eco-friendly products. Similarly, immobilization of heavy metals and radionuclides has been treated *via* this method (Adeleye et al., 2016; Ibrahim et al., 2016; Mondal et al., 2020). Interestingly, the purification of water has been reported by applying MNPs especially targeting bacteria, dye degradation, removal of organic species.

**FIGURE 5:**
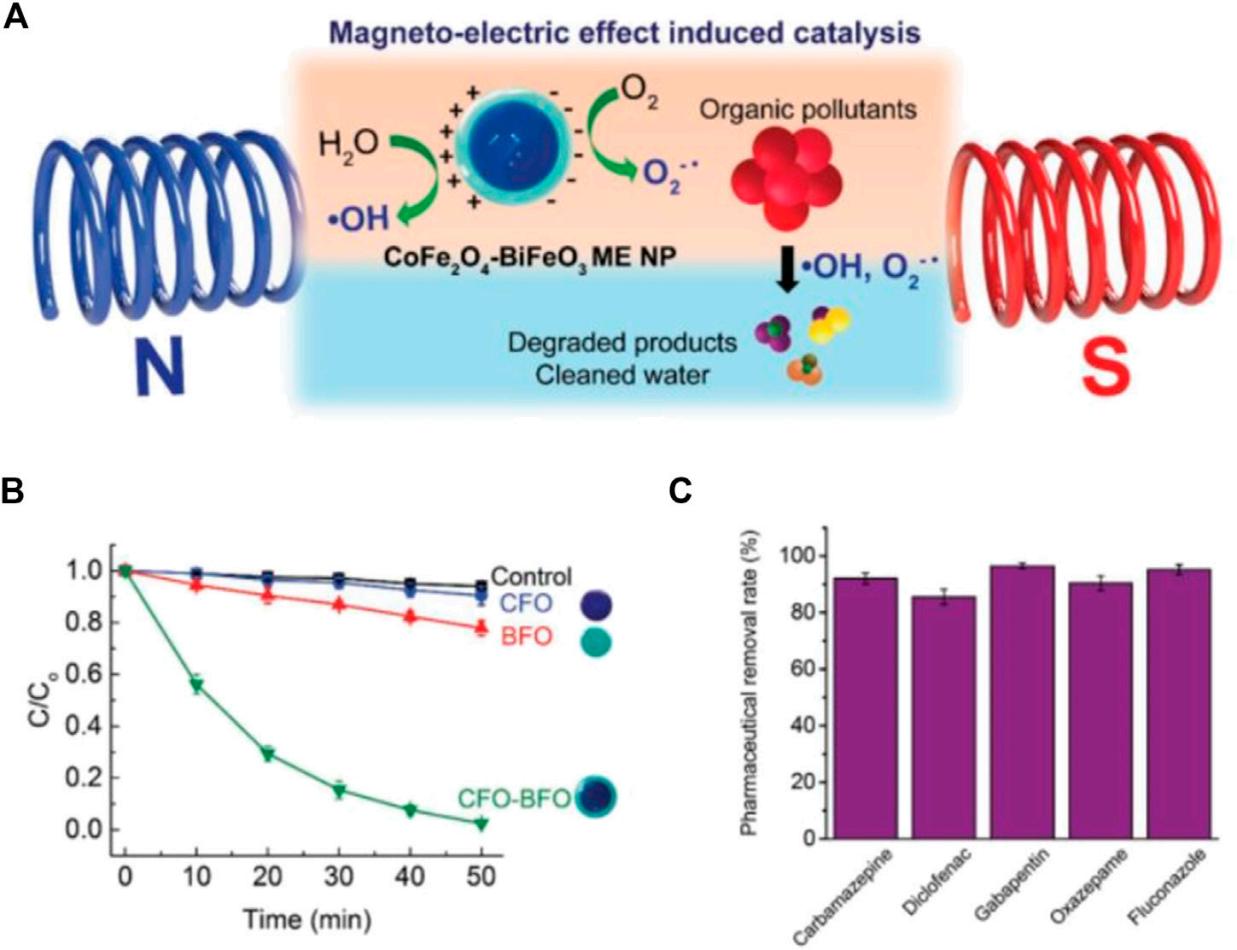
**(A)** Scheme showing magnetoelectric (ME) effect induced catalytic degradation of organic pollutants using core–shell CFO–BFO NPs under magnetic fields **(B)** Catalytic degradation curves obtained for model organic dye, RhB, under 15 mT and 1 kHz magnetic fields (*n* = 5) **(C)** Removal efficiency of a cocktail of five common pharmaceuticals using the core–shell NPs (*n* = 4). Copyright 2019, John Wiley and Sons (Mushtaq et al., 2019).

One of the best examples is Fe_3_O_4_@amino acid for the magnetic separation of contaminants from wastewater. Fe_3_O_4_ NPs surface were modified and functionalized with three different amino acids such as arginine, lysine, and Poly-L-lysine forming Fe_3_O_4_@Arinine, Fe_3_O_4_@Lysine, and Fe_3_O_4_@ Poly-L-lysine. The functionalized Fe_3_O_4_@AA shown greater capturing ability for both gram-positive and negative bacteria such as *Bacillus subtilis* and *Escherichia coli,* respectively. About 97% of the bacterial were captured and removed by using all of the three kinds of Fe_3_O_4_@AA (Jin et al., 2014). Due to its magnetic property, iron oxide serves as cost-effective and has easy separation from aqueous solutions because they aggregate rapidly by applying an external magnetic field (Bhalerao, 2019). Currently, magneto-catalysis has been considered as the most effective method for degrading persistent pollutants/dye under external stimulus-response to degrade pollutants/dye. The underlying mechanism is magnetoelectric induce catalytic degradation of organic pollutants *via* generating free radicals to react with parent compounds by converting them to low-risk compounds. The capability of the MNPs has been investigated by applying the alternating magnetic field to degrade the organic pollutants, rhodamine B (RhB). For example, Pane’s group designed cobalt ferrite–bismuth ferrite (CFO–BFO) core–shell nanoparticles with magnetoelectric feature to catalytically degrade a model organic pollutant, Rhodamine B (RhB) and other pharmaceutical compounds (Mushtaq et al., 2019). They designed a magnetoelectric system with combination of magnetostrictive CFO and multiferroic BFO to purify water through oxidation processes under wireless magnetic fields without assistance of catalytic molecules. The magnetostrictive CoFe_2_O_4_ (CFO) NPs were prepared *via* hydrothermal process and multiferroic shell BiFO_3_ (BFO) was created by sol-gel method. Their finding reveals that as-synthesized NPs through magnetoelectric induction generate hydroxyl and superoxide radical, which catalytically degrade RhB with 97% removal efficiency and a mixture of pharmaceutical micropollutants with 85% removal efficiency (Figure 5). The microbial contamination of the environment is a serious problem. Numerous kinds of microbes as well as toxic ions from the agriculture and industrial waste discharge to water bodies. The MNPs are being employed for the purification of wastewater to remove organic/inorganic pollutants, degrading dyes, and killing/separation of microorganisms.

Previously, different studies reported chemical pollutants like pesticides and antibiotics are degraded by using MNPs with catalytic and photocatalytic properties through oxidation and reduction processes (Hodges et al., 2018). MNPs surface can be functionalized with stabilizers showing great potential for wastewater treatment due to their stability, less aggregation, and large surface area with recycling capability (Xu et al., 2012). Organic pollutants have been removed through the use of superparamagnetic iron oxide NPs (SPIONPs) and their nanocomposites (Kilianová et al., 2013). Various kinds of micropollutants like toxic dyes have been removed *via* nanocomposites. A study reported amine-functionalized magnetite Fe_3_O_4_–SiO_2_–NH_2_ NPs has been synthesized for the removal of viruses and bacteria from the water. Such novel kinds of MNPs have well-established structures and core-shell having good magnetic properties. The amine group in the MNPs has a great deal of attraction for various kinds of pathogens such as bacteriophages, *poliovirus-1*, and bacterial like *P. aeruginosa*, *Salmonella,* and *B. subtilis* (Zhan et al., 2014).

There is limited information about the transfer and availability of iron NPs in the atmosphere. The transport and movement of pure iron NPs have been restricted because of their colloidal nature. The migration of iron NPs has reported only a few feet at the injection point (Lei et al., 2018). Furthermore, the movement of NPs depends on their size, pH, the strength of ions, and the composition of soil or groundwater, velocity, etc. However, it must be noted that the utmost serious criteria such as toxicity and bioaccumulation must be evaluated. It is extremely important to investigate the toxicity mechanism to ensure the biosafety and stability of nanomaterials (Ali et al., 2020). These research gaps need to be well addressed to overcome challenges of water and soil quality. The nano-based treatment processes of wastewater have a great potential to improve environmental quality as compared to conventional methods. These approaches used for cleaning water can reduce the power and energy consumption, use of chemicals, and residual wastes. In this regard, the use of MNPs can play a great role to minimize the risks associated with water cleaning processes. The nanotechnology possesses great potential with unprecedented opportunities in improving water and environmental quality (Alvarez et al., 2018).

### Agriculture

**FIGURE 6:**
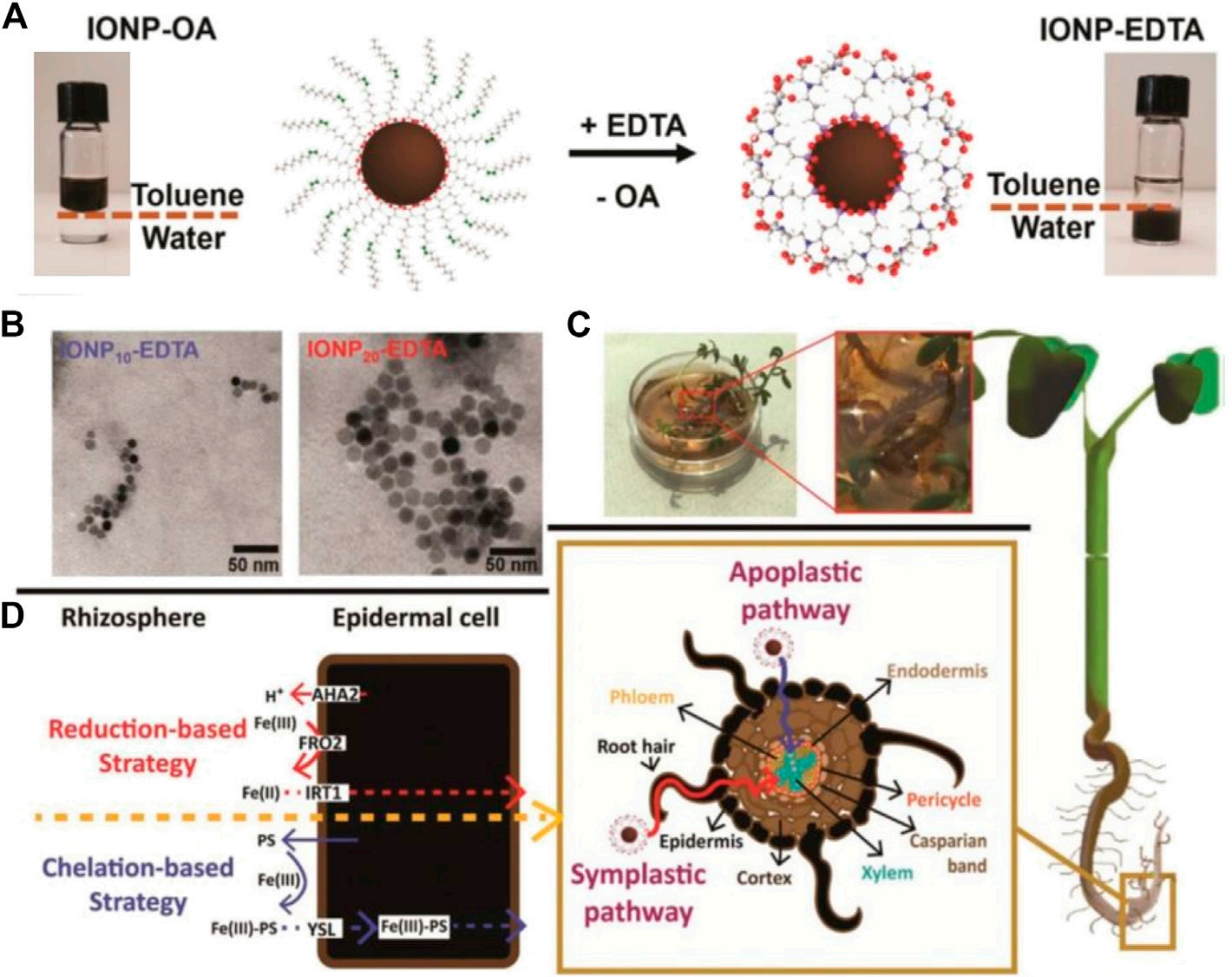
**(A)** Schematic diagram of the ligand exchange process to generate water-soluble EDTA-capped iron oxide nanoparticles (IONP-EDTA) from as-synthesized oleic acid capped iron oxide nanoparticles (IONP-OA) **(B)** TEM images of IONP10-EDTA and IONP20-EDTA after plant incubation in hydroponic growth media **(C)** Representative photograph of IONP-EDTA samples concentrating around the root hairs of garden cress after 30 min of incubation **(D)** Schematic representation of the pathways for the uptake of Fe through the plant roots. Copyright 2019, Royal Society of Chemistry (Ju et al., 2019).

Several researches have been conducted showing the successful application of metallic NPs in plant protection, seed germination, and improving soil quality (El-Temsah et al., 2016; Rui et al., 2016). For example, iron oxide MNPs can be used as soil nutrition to increase production with minimum negative impacts (Mishra et al., 2017). Iron is a very essential element that plays a significant part in numerous physiological activities such as respiration, biosynthesis, chlorophyll, and redox reactions (Rout and Sahoo, 2015). Several crops e.g., peanuts exhibit iron deficiency. In this context, many studies have been conducted to overcome the deficiency and to improve iron consumption with application of iron NPs (Zuo and Zhang, 2011; Sánchez-Alcalá et al., 2014; Cheng et al., 2015; Zia-Ur-Rehman et al., 2018). Mostly, NPs are in hydroponic conditions as nanofertilizers instead in field conditions. Iron is considered the most abundant plant nutrient in the soil. However, its availability remains challenging for plants. Therefore, functionalized iron oxide NPs can overcome this problem as well as overpass barriers and uptake by plants through a analytical processes. In this regard, Ju et al. synthesized iron oxide NPs through the thermal decomposition method and capped with oleic acid (IONP-OA), followed by a ligand exchange process with N-(trimethoxysilylpropyl) EDTA produced water-soluble iron oxide NPs (IONP-EDTA) to monitor iron uptake and transport in plant (Ju et al., 2019). They designed a system to monitor the uptake and distribution of different sizes chelating iron NPs (10 and 20 nm) through magnetic particle spectroscopy. The authors reveal that Fe ions coming from the larger IONP_20_-EDTA NPs was more compared to the smaller IONP_10_-EDTA. There is an increased production of biomass and chlorophyll in the case of IONP_20_-EDTA treatment. The images taken by TEM show no considerable changes in the size or morphology of the particles released either from IONP_10_-EDTA and IONP_20_-EDTA samples. The accumulation of iron oxide NPs takes place in the vicinity of the root and hence shows the uptake strategy *via* roots in the plant. The uptake of Fe has been demonstrated through root *via* different oxidation states and also showing the two routes for the uptake and translocations of the iron oxide NPs into the upper components of the plants (apoplastic and symplastic pathways) as demonstrated in (Figure 6). Even though different MNPs have been used as antimicrobial agents in biomedical research for treating various kinds of diseases while in plants the use of MNPs in disease management is still in infancy (Duguet et al., 2006; Jurgons et al., 2006). MNPs can be helpful for targeted delivery to distinct areas of the plant. it’s very beneficial to track the internal transport of the MNPs for the targeted treatment of specific regions in plants (Arakha et al., 2015). Previously, the transfer of biomolecules into plant cells *via* magnetic NPs and the use of their magnetic characterization to guide carriage and localization has been reported. For example, a study reported *in vitro* culturing of Cucurbita pepo and carbon-coated magnetic iron NPs for plant’s disease treatment. The localization was spotted with confocal, light, and electron microscopy (González-Melendi et al., 2008). The conjugation of MNPs with various biomolecules such as nucleic acids, chemicals, and enzymes is very useful in smart delivery systems. The delivery of a gene and its expression into the host cell is very efficiently used. Currently, the transport of genes is delivered by three kinds of carrier systems such as virus, electroporation *via* nucleic acid, and transfection. The transfection technique holds a very prominent solution for many medical problems. The transfection efficiencies of cultured cells can be improved by coating with MNPs (Kudr et al., 2017). The use of NPs can elevate toxicity inside the cells which restrains their applications in different researches both *in vivo* and *in vitro*.

### Catalysis

**FIGURE 7:**
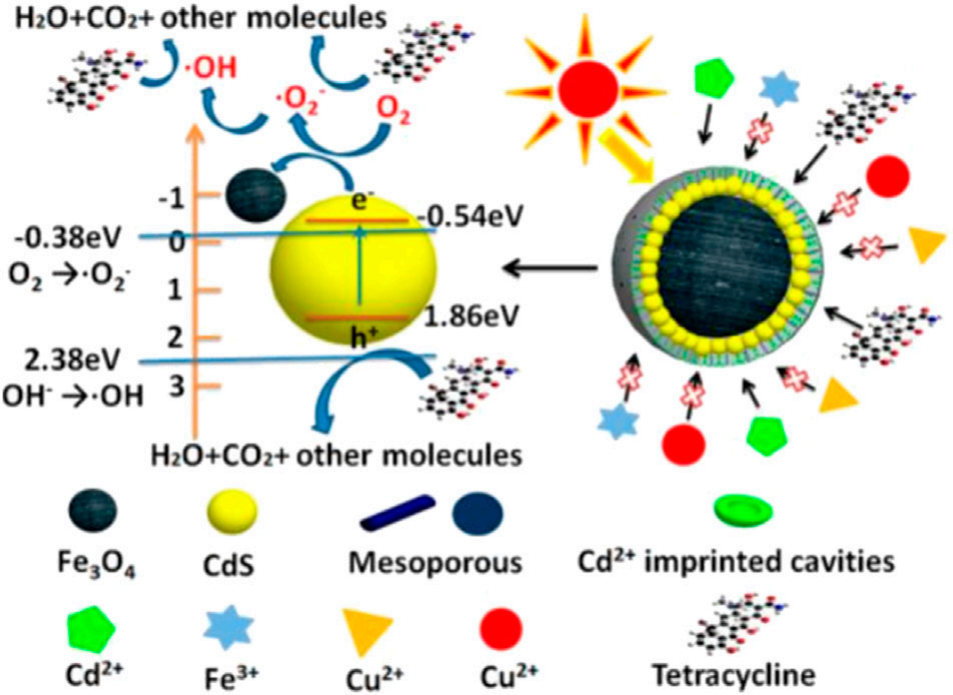
Proposed photocatalytic and selective mechanism of the MHP-Cd. Copyright 2019, American Chemical Society (Lu et al., 2019).

Until now various catalytic processes and systems have been established for the conversions of reactants to products (Liu and Zhang, 2016). One of the limitations of homogeneous catalysts is the separation difficulty from the reactions. Recently the limitation of heterogeneous catalysis has been reduced and minimized using catalysts that are supported by MNPs. The MNPs have the ability that they provide a high surface area to support active sites for reactants to be converted into products easily while the separation of such catalysts combined the advantage of high dispersion and reactivity (Lee et al., 2008). In heterogeneous catalytic reactions, magnetic materials with good reproducibility have been reported (Martínez-Edo et al., 2018; Sudarsanam et al., 2018; Zuliani et al., 2018). Recently, the photocatalytic system emerged as an efficient and reliable method for pollutant degradation under natural light. In this system, sunlight is used as an external stimulus source to activate the system and generate free radicals that react with pollutants leading to degradation. In this regard, Xing’s group developed light responsive magnetic hierarchical porous cadmium (Cd^2+^) imprinted photocatalytic nanoreactors (MHP-Cd) with excellent adsorption capacity for tetracycline degradation (Lu et al., 2019). Their findings reveal uniform dispersion of MHP-Cd in Cd^+2^ solution allowed selective adsorption of Cd^+2^ in the cavities by keeping Cu^2+^, Fe^3+^, and Zn^2+^ outside. After the addition of MHP-Cd in tetracycline solution followed by exposure to sunlight, the Cds absorbed the light and excited free electrons (e^−^) and heat energy (h^+^). The generation of one e^−^ in the Cds helps in the transformation of Fe_3_O_4_. While another e− was used by the O_2_ to generate superoxide (•O^2−^) and hydroxyl (•OH) radicals. Due to excellent affinity a greater amount of tetracycline arrived through the mesoporous channels at Cds surface and transformed into carbon dioxide (CO_2_), water (H_2_O), and smaller molecules (Figure 7). In addition, there is much need to develop and established suitable strategies to control and tuning the particle size and form by different synthetic approaches. It is still prevalent to design and establish the highly stable and robust MNPs for industrial applications. Such kinds of NPs must be scalable and economical that can resist the reaction conditions in heterogeneous reactions (Deng et al., 2017).

## Challenges and Future Perspectives

MNPs are being applied in different fields such as biomedical, environmental, agriculture, and catalysis and biosensing. In this review, we summarized recent advances in the synthesis, characterization and the potential applications of MNPs. Different kinds of MNPs showing some promising properties are being produced by using different synthetic methods. These methods include ball milling, thermal decomposition, hydrothermal synthesis, microemulsion synthesis, gas-phase condensation, sol-gel, biological synthesis method, etc. For the synthesis of the MNPs in large amount physical method such as the ball milling are being used, however, this method poses contamination from the milling jars and balls. While on the other hand, thermal decomposition or pyrolysis method is used to produce monodisperse MNPs. The pyrolysis method has advantages because of its simple and good control over the size of MNPs. The sol-gel synthesis method are used produce MNPs with uniform size distribution and superior stoichiometric control at low processing temperature. The aforementioned methods propose various kinds of MNPs for important biological and biomedical applications. Human development has been hindered by various kinds of problems such as cancer, pollution, agriculture practices, and so on. To address these problems different kinds of functionalized NPs have been developed in past decades. Nano-based cancer therapy mainly depends upon the efficient and smart design of NPs, by treating cancer more safely and effectively. In Recent decades, MNPs have made a greater contribution to nanomedicines due to their unique characteristics. Currently, various kinds of MNPs modalities have been under clinical investigations for cancer cell imaging and therapies. MNPs synthesis and formulations face critical biological barriers, such as localization at the target site, the effective delivery of the drug to the target site, cross physiological talk, and the other technical obstacles specific to cancer. Some other kinds of barriers are clearance, endosomal escape, off-target sites, and drug efflux. The resistance of bacteria against widespread antibiotics has become a serious health concern in both developing and developed countries. Due to various kinds of multidrug-resistant strains, emergence and the unavailability of new antibiotics will cause a serious threat. In the current decade, MNPs based strategies have been developed to treat infections caused by pathogenic microbes and eradicate the biofilms with minimum resistance. The MNPs have great importance and are widely used in targeted drug delivery. As compared to conventional drug delivery, MNPs based drug administration can minimize the doses of drugs and subsequently reduce the side effects. Additionally, the MNPs have inherent antimicrobial activity, by combining these antimicrobial molecules with them has synergistic effects in therapies which lead to improving the efficacy of antimicrobial drugs. Similarly, the purification of wastewater are being practiced by using MNPs including removal of organic/inorganic pollutants, dyes degradation, killing or separation of microbes. The MNPs can be used as soil fertilizers to increase production as well as plant disease management. The conjugation of MNPs with various biomolecules such as nucleic acids, chemicals, and enzymes is of great use in research. In this regard, the delivery of a gene and its expression into the host cell is efficiently used. The MNPs show tremendous potential in heterogeneous catalysis as well as in other applied fields. The coating of the catalysts with magnetic NPs have been used in different kind of catalysis and clean energy. The MNPs can provide numerous active sites for the reactants to be converted into products.

To overcome challenges, critical and constructive research are demanded to design and fabricate MNPs for diverse application in different fields. With the advancement and to make multidisciplinary approaches it must be considered to build regulatory institutions for the safe and effective use of nanotechnology. A regular mechanism and connection must be conducted between the institutions and researchers to develop specific standards and platforms to escalate clinical trials and pre-clinical studies *in vivo*. MNPs face an enormous number of challenges to be practically implemented for the treatment of cancer and to combat multidrug resistance. The ratio of magnetic NPs and catalysts must come under discussion. One of the big challenges is the biocompatibility and the toxicity of the MNPs in long term. A very detailed and comprehensive study must be conducted to study the composition, morphology, size, shape, structure, and side effects of MNPs. The scientific community needs to address such kind of enormous challenges and has to conduct hassle-free clinical trials for the development and construction of MNPs for a better future.
